# Establishing simple image-based methods and a cost-effective instrument for toxicity assessment on circadian rhythm dysregulation in fish

**DOI:** 10.1242/bio.041871

**Published:** 2019-06-10

**Authors:** Gilbert Audira, Bonifasius Putera Sampurna, Stevhen Juniardi, Sung-Tzu Liang, Yu-Heng Lai, Liwen Han, Chung-Der Hsiao

**Affiliations:** 1 Department of Chemistry, Chung Yuan Christian University, Chung-Li 32023, Taiwan; 2 Department of Bioscience Technology, Chung Yuan Christian University, Chung-Li 32023, Taiwan; 3 Department of Chemistry, Chinese Culture University, Taipei 11114, Taiwan; 4 Biology Institute, Qilu University of Technology (Shandong Academy of Sciences), Jinan City, Shandong, China; 5 Center of Nanotechnology, Chung Yuan Christian University, Chung-Li 32023, Taiwan; 6 Center of Biomedical Technology, Chung Yuan Christian University, Chung-Li 32023, Taiwan

**Keywords:** Circadian rhythm, IdTracker, ImageJ, Locomotion

## Abstract

Analysis of circadian rhythm behavior alteration in fish for toxicity assessment usually requires expensive commercial equipment and laborious and complicated tweaking. Here, we report a simple setup that consists of a custom-made light box equipped with white and 940 nm light-emitting diode (LED) light strips as light sources, where the locomotion activities of zebrafish or catfish are captured using an infrared-sensitive coupled charged device (CCD). The whole setup was housed in a temperature-controlled incubator to isolate external noise and to maintain consistent experimental conditions. The video recording and light triggering were synchronized using Total Recorder, a recording scheduling software. By using the setup mentioned above and open source software such as ImageJ or idTracker, the locomotion activities of diurnal (e.g. zebrafish) and nocturnal (e.g. catfish) fish during day and night cycles can be quantitatively analyzed. We used simple image-based methods and a cost-effective instrument to assess the circadian rhythm of multiple fish species, as well as other parameters such as age, ambient temperature and chemical toxicology with high precision and reproducibility. In conclusion, the instrument setting and analysis methods established in this study provide a reliable and easy entry point for toxicity assessment on circadian rhythm dysregulation in fish.

## Abstract (precis)

**Summary:** A cost-effective device and two methods to measure the circadian rhythm in diurnal and nocturnal fish, providing a reliable entry point for toxicity assessment on circadian rhythm dysregulation in fish.

## INTRODUCTION

Circadian rhythms are based on intracellular time-tracking systems that play a central role in adapting the physiology and behavior of living organisms to anticipate daily environmental changes (Lahiri et al., 2005). The principal cue that influences circadian rhythmicity is light (Hirayama et al., 2005). Circadian rhythms are driven by endogenous circadian clocks that are comprised of self-sustained oscillators that drive rhythms within a period near 24 h in the absence of external timing cues. Circadian clocks involve *clock* genes that interact to produce a molecular oscillator adjusting output clock-controlled genes. Thus far, some *clock* genes have been identified in vertebrates and most of the studies on the molecular mechanisms of circadian rhythm generation have come from research of *clock* genes that were originally identified by mutations, which altered or abolished circadian rhythms (Delaunay et al., 2000). To date, many circadian rhythm mutants have been discovered in *Arabidopsis*, *Drosophila*, *Neurospora* and *Synechococcus.* However, only a handful of circadian genes have been discovered in vertebrates.

In the past few years, the use of zebrafish (*Danio rerio*) has been expanding from its traditional user base to studying various biomedically relevant aspects of biology, including behavior and physiology. The rapid accumulation of zebrafish genetic and genomic information also makes zebrafish potentially useful vertebrates for mutational analysis of vertebrate circadian rhythmicity, especially in circadian gene regulation and functions of the pineal organ (Gamse et al., 2002). The zebrafish circadian system shares some similarities with that of *Drosophila*, and their tissues contain endogenous circadian oscillators that are light responsive and entrainable to light–dark (LD) cycles *in vitro* (Tamai et al., 2007). Even though the interest in zebrafish as a vertebrate model organism does not focus on the circadian clock, several aspects of their basic biology make them inherently ideal for this area of study. First of all, their embryos are completely transparent and develop rapidly in an egg shell or chorion, so the whole developmental process can be observed non-invasively under the microscope. Secondly, their reproduction is relatively simple and they can be raised at high density at low cost. In addition, adult zebrafish are hardy, small (2–3 cm long) and reach sexual maturity after only 2–3 months. Another advantage is that zebrafish are ideal for large-scale forward and reversed genetic screening (Vatine et al., 2011). Because of their potential usefulness for circadian rhythmicity research, efficient methods for analysis of circadian rhythm phenotypes in zebrafish are needed (Cahill et al., 1998).

**Fig. 1.**
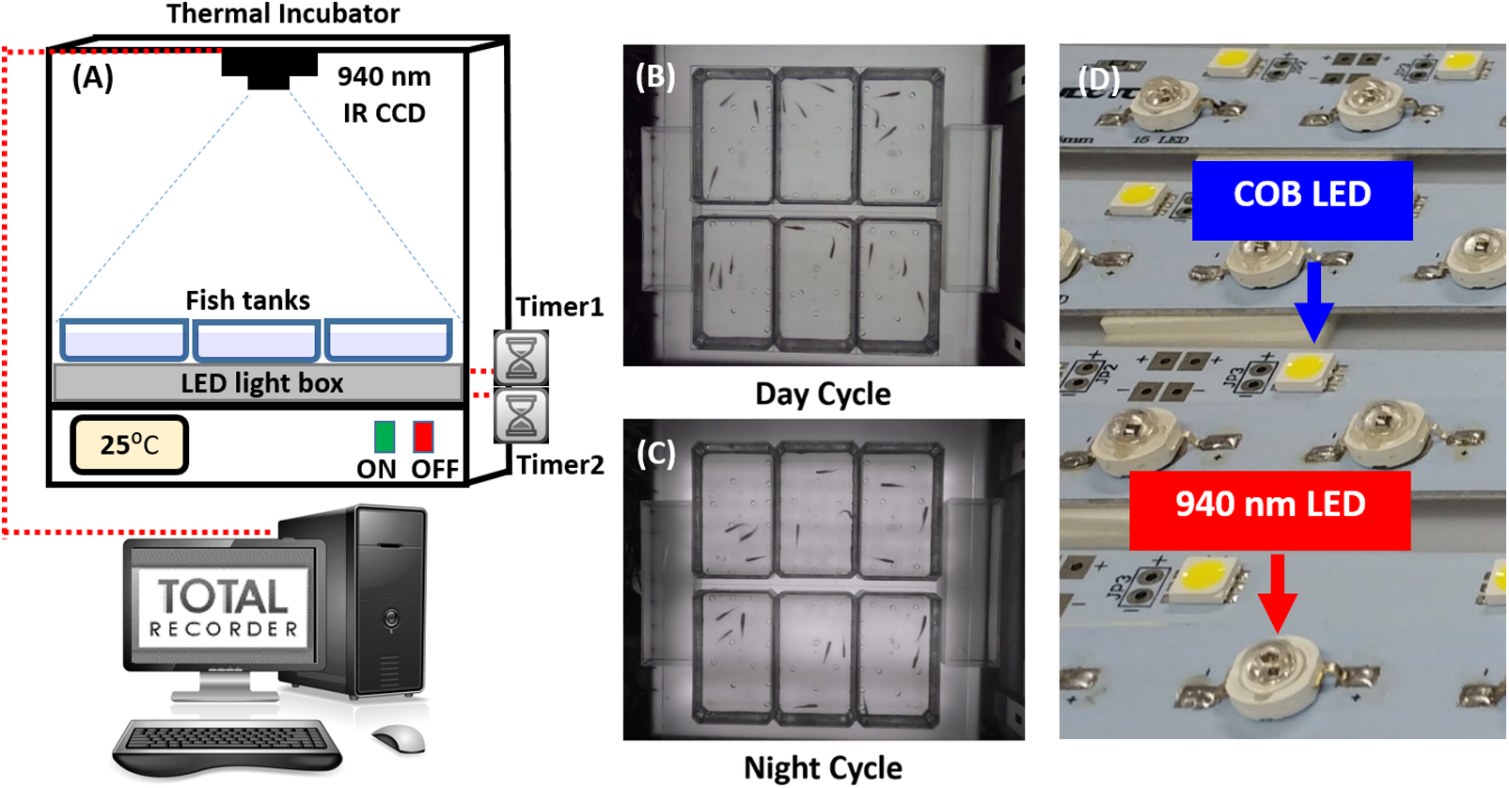
**Overview of the custom-made light box chamber to detect the circadian rhythm in fish.** (A) Schematic of the fish circadian rhythm chamber, which consisted of an IR camera (IR CCD), an LED light box and a temperature control incubator. (B,C) Example snapshots of fish position in the water tank during the (B) light (day) and (C) dark (night) cycles. (D) LED light source arrangement for the fish circadian rhythm chamber.

Maintained circadian locomotor activity rhythms of animals in constant conditions have been utilized to study the functions of some *clock* genes and to screen for circadian clock mutants (Antoch et al., 1997; King et al., 1997; Konopka and Benzer, 1971; Ralph and Menaker, 1988). These locomotor rhythms have also been used comprehensively to analyze the physiological organization of mammalian, reptilian and avian circadian systems. However, few studies of teleost locomotor rhythmicity in constant conditions can be found in the literature, even though current available data reveal considerable variability in the strength and pattern of teleost locomotor activity rhythms. To date, there have been few reports of circadian behavioral rhythmicity in zebrafish (Hurd et al., 1998). Several techniques for the characterization of behavioral circadian rhythmicity in zebrafish have been developed. However, most of the assessments were conducted in zebrafish larvae and the activity records were analyzed manually (Cahill et al., 1998; Hurd and Cahill, 2002). Moreover, using either larvae or adult zebrafish, the obtained data cannot describe their precise spatial information (Hurd et al., 1998; Zhdanova et al., 2008). In addition, we also tested our method in Indian walking catfish (*Clarias batrachus*). These important commercial species of fish were used because they are nocturnal animals and their circadian rhythm of locomotor activity can help to validate the usability of our current method. However, even though the catfish is a nocturnal animal, it exhibits LD-entrainment of its locomotor activity that free-runs under constant conditions. It appears that the overt circadian rhythm of locomotion in this fish is underlain by multiple oscillators (Ramteke et al., 2009). Therefore, acclimatization for these fish to the current photo-regime condition is mandatory before the experiment. Overall, in this study, we aim to develop an automatic and reliable circadian activity measurement method and related instrument for zebrafish and other fish species (Fig. 1).

## RESULTS

### Measurement of the circadian rhythm in zebrafish and catfish

**Fig. 2.**
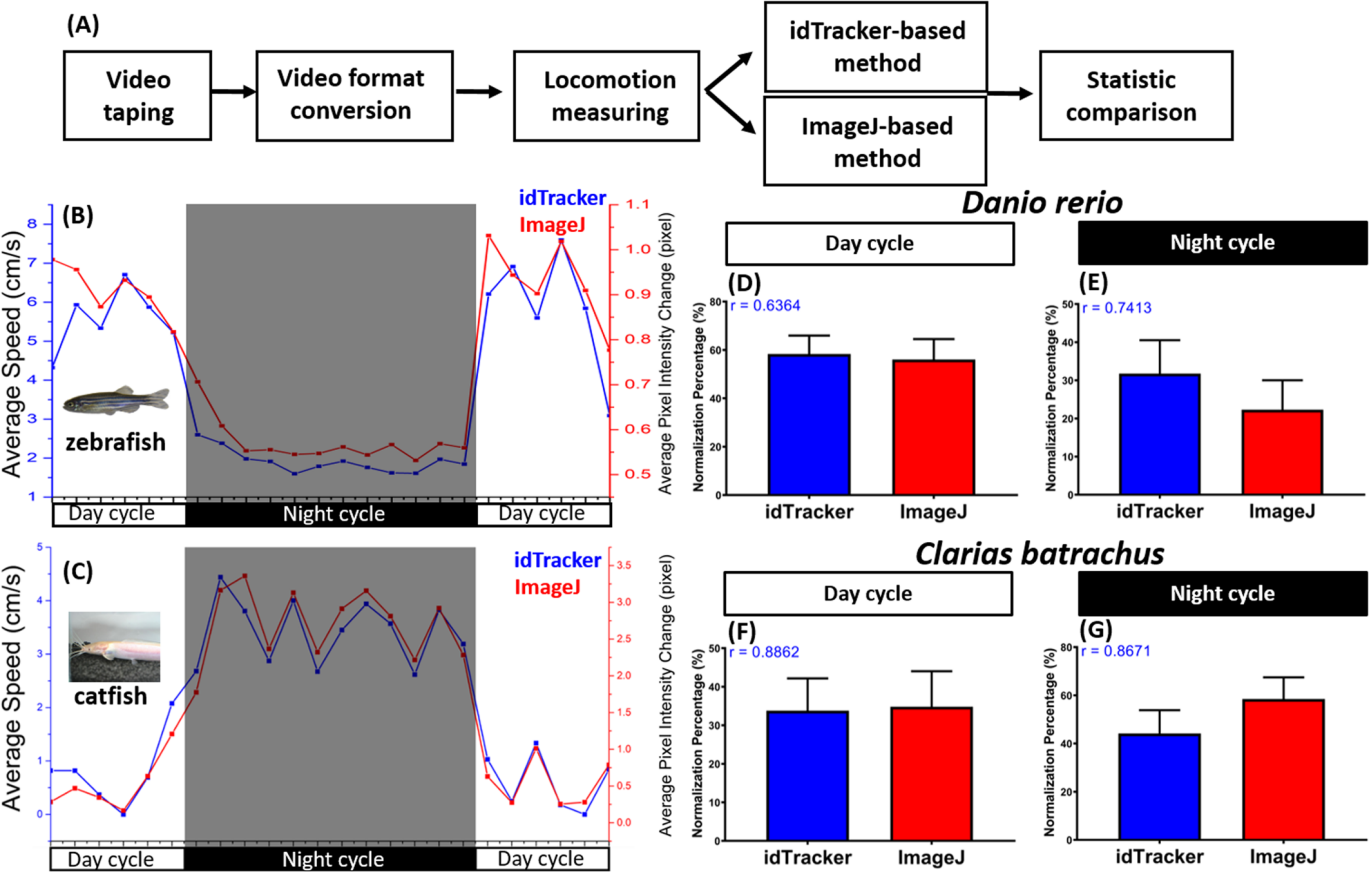
**Comparison of circadian rhythm locomotion activities of zebrafish and catfish measured by the idTracker or ImageJ methods.** (A) Schematic of the analysis pipeline for fish locomotion measurement in this study. (B,C) The circadian rhythm of diurnal fish species (B, zebrafish) and nocturnal fish species (C, catfish) identified by either idTracker (blue) or ImageJ (red). The data are expressed as the means. (D,E) Comparison of normalized percentages between idTracker and ImageJ results after data normalization in total zebrafish circadian activity in (D) day cycle and (E) night cycle with *r-*value (highlighted in blue) from Spearman’s nonparametric correlation test. (F,G) Comparison of normalized percentages between idTracker and ImageJ results after data normalization in total catfish circadian activity in (F) day cycle and (G) night cycle with *r-*value (highlighted in blue) from Spearman’s nonparametric correlation test. The data are expressed as the means±s.e.m. (zebrafish, *n*=18; catfish, *n*=6) and were analyzed by unpaired *t*-test with Welch's correction.

To explore the potential utility of these two protocols, we validated the method by using diurnal (zebrafish, *D.* *rerio*) and nocturnal (catfish, *C*. *batrachus*) fish species with distinct locomotion activities during the light and dark cycles. For zebrafish, we found robust swimming activity during the light cycle and a sleep-like behavior during the dark cycle (Fig. 2B and Movie 1). This was exemplified by higher than average speed and rapid movement–time ratio during the light cycle compared to the dark cycle (Fig. S1D,E). Furthermore, this behavior was also demonstrated by the low level of meandering and freezing time–movement ratio exhibited by zebrafish during the light cycle (Fig. S1F,G). On the other hand, catfish were active in the dark cycle and showed a sleep-like behavior during the light cycle (Fig. 2C and Movie 2). This behavior was indicated by a lower than average speed and rapid movement–time ratio during the light cycle compared to the dark cycle (Fig. S1H,I). In addition, high levels of freezing time–movement ratio and meandering were observed in this experiment (Fig. S1J,K). This result is in line with the literature showing most catfish species have nocturnal behaviors (Vera et al., 2011; Ramteke et al., 2009). The locomotor activity of catfish is entrained by light–dark cycles. Catfish activity increased during the dark periods, indicating that catfish are nocturnal. However, zebrafish locomotor activity intensified during the light periods, showing that zebrafish are diurnal (Kasai et al., 2009). For both fish species with distinct circadian rhythms, we cross-examined the locomotion activity curves acquired by idTracker- and ImageJ-based methods. After data normalization, an unpaired *t*-test with Welch's correction was used to analyze the differences between these data. Later, we found that both data were paralleled and correlated with each other, indicating that both methods can be used to measure fish circadian rhythm activities with high reliability and reproducibility. Furthermore, from the *r-*values that we obtained from Spearman's nonparametric correlation test, we concluded that the two variables (ImageJ and idTracker results) tend to increase or decrease together, or in other words, they correlate with each other, with high *r-*values supported (Fig. 2D,E for zebrafish and Fig. 2F,G for catfish).

### Measurement of the circadian rhythm in zebrafish with different age and temperature

**Fig. 3.**
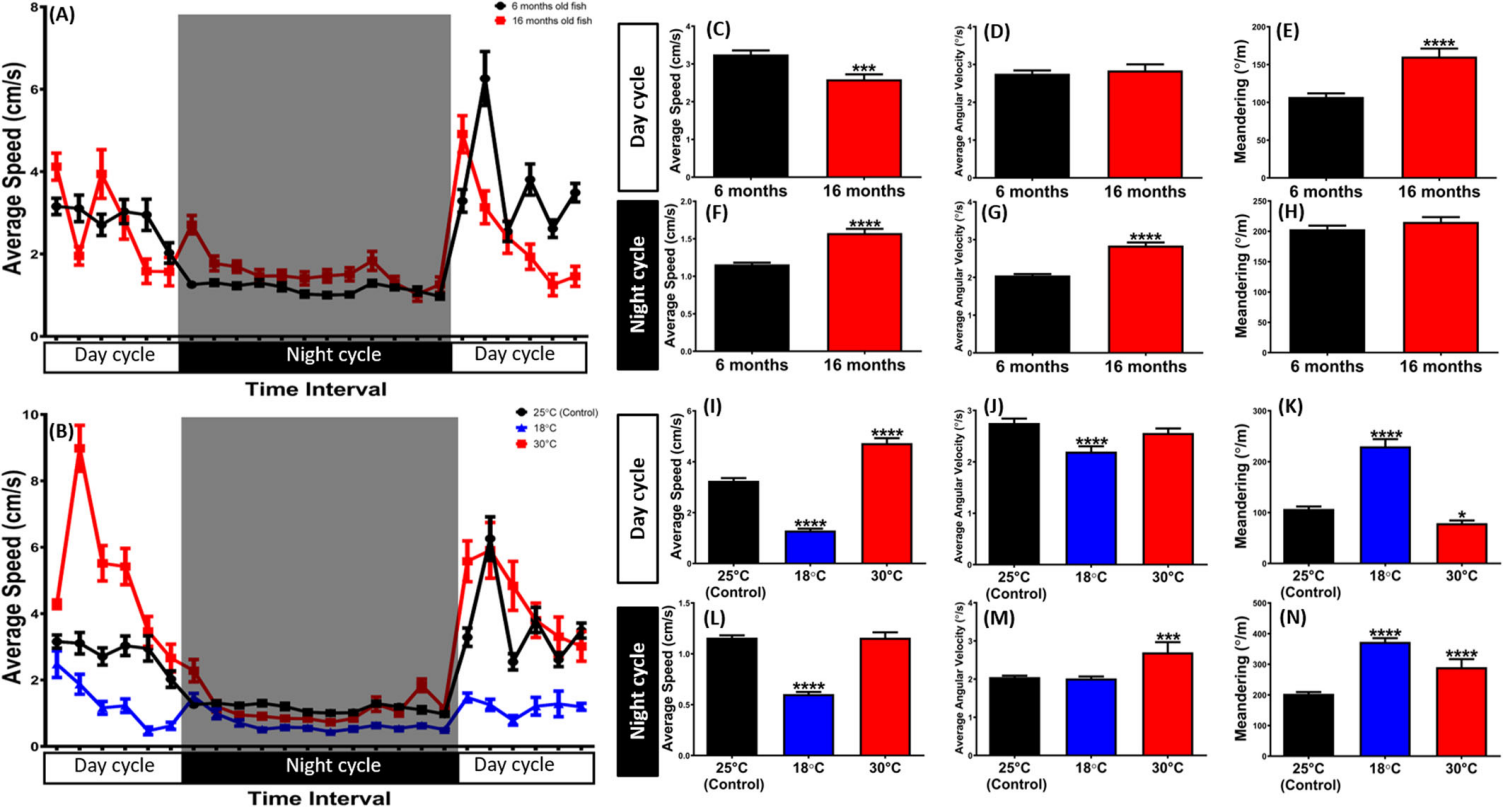
**Effects of different ages and temperatures on circadian rhythm locomotion activities of zebrafish.** (A) Comparison of the circadian rhythm locomotion activities between 6-month-old (black) as a control and 16-month-old (red) zebrafish. (B) Comparison of the circadian rhythm locomotion activities for zebrafish acclimated to either ambient temperature at 18 (blue), 25 (black) or 30°C (red). (C–H) Comparison of average speed (C,F), average angular velocity (D,G) and meandering (E,H) of zebrafish between the old fish and control fish during the light and dark cycles. The data are expressed as the means±s.e.m. (control, *n*=28; old fish, *n*=18) and were analyzed by unpaired *t*-test. (I–N) Comparison of average speed (I,L), average angular velocity (J,M) and meandering (K,N) of zebrafish when they incubated in 18 (blue), 25 (black) or 30°C (red) during the light and dark cycle. The data are expressed as the means±s.e.m. (18°C, *n* =13; 25°C, *n*=28; 30°C, *n*=18) and analyzed by Dunnett's multiple comparisons test. All of the multiple comparisons tests compared the mean of each column with the mean of a control column. Significance difference was defined as *P<0.05, ***P<0.001, ****P<0.0001.

Aging is the accumulation of deleterious changes over multiple levels of biological organization resulting in a decrease in whole-organism functionality over time (Gilbert et al., 2014). The results of multiple studies in humans and animal models suggest that aging alters the circadian clock (Zhdanova et al., 2008). In order to study the age effect in zebrafish circadian rhythm, we measured the circadian rhythm locomotion activities for 6-month-old (*n*=28) and 16-month-old (*n*=18) zebrafish. We found that the older zebrafish showed higher locomotion in the dark cycle and lower locomotion in the light cycle compared to younger zebrafish (6-month-old) (Fig. 3A, Fig. S1A and Movie 2). This phenomenon is supported by a low level of average speed and a high level of meandering in the light cycle (Fig. 3C,E) and high levels of both average speed and average angular velocity in the dark cycle (Fig. 3F,G). Meanwhile, there was no difference in average angular velocity during the light cycle and meandering during the dark cycle between the adult fish and the old fish (Fig. 3D,H). This result is in agreement with a previously published study showing that zebrafish aging is associated with significant changes in rhythms of activity, sleep and melatonin production (Zhdanova et al., 2008). In the dark cycle, reduced sleep-like behavior was exhibited by the older tested zebrafish. Sleep-like behavior in zebrafish is classified as prolonged periods of inactivity (over 5 s). During this period, zebrafish may stay motionless or occasionally exhibit very slow movement. Melatonin, a hormone produced in the pineal gland, induces sleep in some contexts and plays a key role for mediating the circadian process because *clock* regulates its production (Gandhi et al., 2015). Melatonin promotes sleep in diurnal animals, including humans and zebrafish, and its production in the middle of the dark phase gradually declines with age. Therefore, reduced hours of sleep time is found in older people, as well as an incrementing number of awakenings and a reduction in the deep-sleep stage of non-REM. Several studies also report that older humans have difficulty maintaining sleep on a regular basis (Duffy et al., 2015; Dijk et al., 2001; Ehlers and Kupfer, 1989; Webb, 1982).

Temperature is considered the ‘abiotic master factor’ among physical factors that can influence behavior, physiology and distribution of aquatic organisms in the aquatic environment. Due to its importance, thermal tolerance has been extensively studied in fishes (López-Olmeda and Sánchez-Vázquez, 2011). For potential temperature effects in the circadian rhythm of zebrafish, we measured the circadian rhythm locomotion activities of 6-month-old zebrafish in ambient temperatures of either 18°C (*n*=13), 25°C (*n*=28) or 30°C (*n*=18) and found there were significant locomotor activity differences in their circadian rhythm (Fig. 3B, Fig. S2A,B and Movie 3). When the temperature was changed from 25°C to 18°C, the activity of zebrafish during the light and dark cycles decreased. In the light cycle, this behavior was indicated by lower than average speed and angular velocity, and a high level of meandering (Fig. 3I–K). Meanwhile, lower than average speed and higher meandering than the control fish in the dark cycle also supported this phenomenon (Fig. 3L,N). In addition, there was no difference in average angular velocity in the dark cycle between the low and normal temperature conditions observed in the tested zebrafish (Fig. 3M). In line with a previous study by Lopez et al., total activity of zebrafish during the day decreased to about 30% when the constant temperature was reduced (López-Olmeda et al., 2006). Furthermore, high temperature also can alter zebrafish circadian rhythm behavior, especially in the light cycle. This alteration, demonstrated by higher than average speed and low level of meandering, indicates hyperactivity behavior (Fig. 3I,K). Meanwhile, the same level of average angular velocity between normal-and high-temperature zebrafish was found (Fig. 3J). In addition, a higher temperature also slightly altered zebrafish behavior in the dark cycle, demonstrated by higher than average angular velocity and meandering compared to the normal temperature as control (Fig. 3M,N and Movie 3). No significant difference was found in average speeds of fish during the dark cycle (Fig. 3L). These findings are in agreement with those of a previous work by McClelland et al., which reported an increase in the maximum sustained swimming speed in zebrafish reared at 28°C. Lopez et al. also found that zebrafish showed higher than average daily activity when they were maintained at high temperature (López-Olmeda et al., 2006; McClelland et al., 2006). Supporting these phenomena, another study already found that temperature had a significant effect on the proportion of animals that exhibited statistically significant rhythmicity. It could be one of the environmental conditions that affects coupling among oscillators; temperature could modulate factors that are not related to circadian rhythms. For example, low or high temperatures could suppress or induce activity and mask the endogenous rhythm of locomotor activity (Hurd et al., 1998). Later, circadian rhythm patterns for age- and temperature-effect groups were analyzed by idTracker and ImageJ and were combined and compared (Fig. S2A,B). In order to cross-examine the locomotion activity data acquired by these two methods, data normalization and statistical analysis were conducted and, later, we found that all data were paralleled and correlated with each other. This finding is also supported by *r-*values that we obtained from Spearman's correlation test, which concluded that these two variables (ImageJ and idTracker results) have a tendency to increase or decrease together (Fig. S1B,C for age effect and Fig. S2C–F for temperature effect).

### Measurement of the circadian rhythm dysregulation in zebrafish after ethanol exposure

**Fig. 4.**
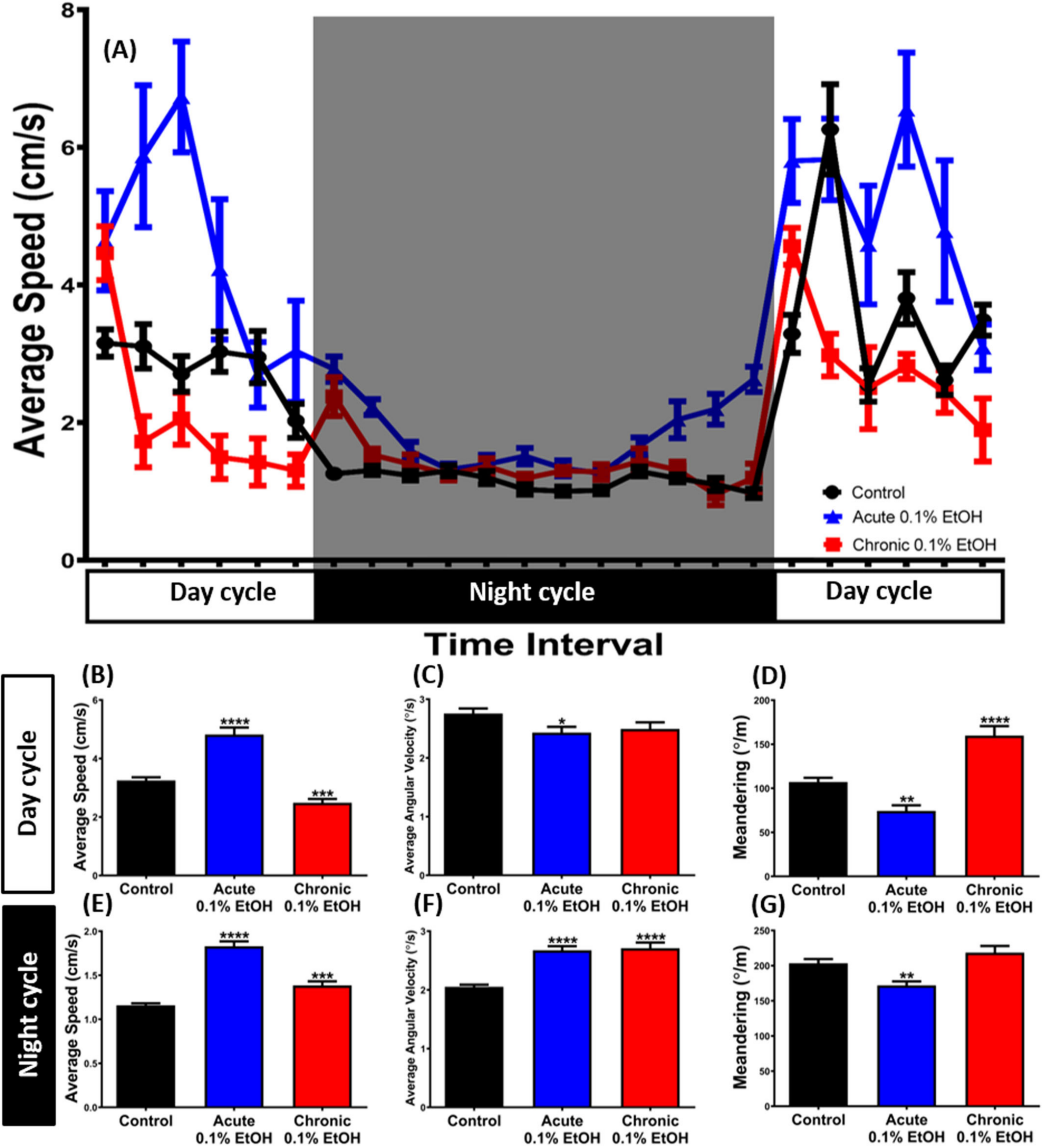
**The circadian rhythm locomotion activities of zebrafish exposed to acute and chronic 0.1% EtOH.** (A) Comparison of the effects of acute (∼30 min, blue) and chronic (∼7 days, red) treatments with 0.1% EtOH on zebrafish circadian rhythm locomotion activities. (B–G) Comparison of average speed (B,E), average angular velocity (C,F) and meandering (D,G) between acutely-treated, chronically-treated and control fish during the light and dark cycle. The data are expressed as the means±s.e.m. (control, *n*=28; acute, *n*=18; chronic, *n*=18) and were analyzed by Dunnett's multiple comparisons test. All of the multiple comparisons tests compared the mean of each column with the mean of a control column. Significance difference was defined as *P<0.05, **P<0.01, ***P<0.001, ****P<0.0001.

Alcohol intake and chronic alcoholism in humans are associated with disruptions of sleep–wake cycles and other daily biological rhythms. Similar disruptions also have been seen in experimental animals treated with chronic ethanol (EtOH) (Rosenwasser et al., 2005). To explore the effects of acute and chronic EtOH ingestion on circadian activity in the zebrafish, we measured the circadian rhythm locomotion activities of 6-month-old zebrafish exposed to acute (∼30 min) and chronic (∼7 days) 0.1% EtOH. Alcohol mixed in the water of the test tank was absorbed by the blood vessels of the skin and the gills of the fish, therefore blood-alcohol levels reached equilibrium with the external alcohol concentration quickly (Gerlai et al., 2000). We found that acute and chronic administration of low concentrations of EtOH altered circadian rhythm activity in both light and dark cycles (Fig. 4A, Fig. S3A,B and Movie 4). In the acute treatment of EtOH, this alteration was indicated by hyperactive behavior, demonstrated by higher than average speeds than control fish in both cycles (Fig. 4B,E). Furthermore, lower than average angular velocity and meandering in acutely-treated fish in the light cycle showed that the fish were infrequently changing their swimming direction (Fig. 4C,D). In addition, sleep disruption caused by acute administration of EtOH was also observed in this experiment, exemplified by similar patterns of swimming direction between the acutely-treated fish in the dark cycle and control fish in the light cycle (Fig. 4F,G). Chronic exposure of 0.1% EtOH, on the other hand, reduced fish activity during the light cycle. This is hypoactivity behavior, indicated by low levels of average speed and high levels of meandering (Fig. 4B,D). Meanwhile, there was no significant difference in average angular velocity between the controls and chronically EtOH-treated fish during the light cycle (Fig. 4C). Similar to acute treatment of EtOH, sleep impairment was also found during the dark cycle in the chronically treated-fish. This phenomenon was indicated by higher than average speed and angular velocity in EtOH-treated fish compared to the control fish (Fig. 4E,F). In addition, there was no significant difference in the meandering for the chronically EtOH-treated fish during the dark cycle (Fig. 4G). These results are in agreement with previous studies about the effect of acute EtOH administration to zebrafish activity; at lower concentrations (e.g. 0.25% and 0.50%), EtOH will increase zebrafish locomotion activity and it will depress their activity at a higher dose (1.00%) (Gerlai et al., 2000). Another study by Lockwood et al. in larval zebrafish showed that they exhibit acute sensitivity to EtOH in a dose- and time-dependent manner. Firstly, they initially become hyperactive and as EtOH accumulates, they become hypoactive and sedated, which is similar to what has been observed in humans and other animal models (Lockwood et al., 2004). These prior findings may explain the hyperactivity behavior in the acute EtOH-treated fish and hypoactivity behavior in the chronic EtOH-treated fish during the light cycle. However, there is no study about the effect of EtOH treatment on circadian rhythm activity in adult zebrafish published until now. The rat study by Rosenwasser et al. showed that both EtOH withdrawal and EtOH intake alter the period and amplitude of free-running circadian activity rhythms (Rosenwasser et al., 2005). Another study in rat also found that chronic EtOH consumption impaired the circadian rhythm of proopiomelanocortin (POMC) and the clock-governing rat period genes mRNA expression in the hypothalamus (Chen et al., 2004). These pilot studies support our results about sleep disorder caused by acute or chronic administration of 0.1% EtOH. Furthermore, in this experiment we found that acute exposure of 0.1% EtOH was more severe than the chronic one during the dark cycle. This phenomenon may be caused by an adaptation ability of zebrafish to EtOH. This statement is supported by another study that provides evidence of adaptation in adult zebrafish to EtOH, showing that after a 2-week long chronic exposure to EtOH, treated zebrafish behaved the same way as the fish that were never treated with EtOH (Gerlai et al., 2006). Afterwards, we cross-examined the locomotion activity patterns for both EtOH exposure groups, analyzed by idTracker and ImageJ methods. Statistical analyses were conducted after data normalization and later, we found that that both sets of data were parallel and correlated each other. The *r-*values from Spearman’s nonparametric correlation test also showed that the two variables (ImageJ and idTracker results) correlate with each other (Fig. S3C,D for acute 0.1% EtOH and Fig. S3E,F for chronic 0.1% EtOH).

### Comparison of instrument settings between previous and current study for circadian rhythm measurement

In rodents, the circadian rhythm can be measured by counting how often they interrupt the infrared (IR) light (Clarke et al., 1985) or microwave signals (Genewsky et al., 2017) in the mouse cage. In catfish, a similar IR-based method has been utilized to analyze catfish circadian rhythm and has found that the catfish is a nocturnal species (Ramteke et al., 2009). The sensitivity of the IR sensor-based method largely relies on the number of sensors; movement events are counted once if the IR signals are interrupted. The IR-sensor method is unable to measure detailed parameters like moving velocity, turning angle and meandering. In this study, by using an idTracker-based approach, we were able to measure these diverse locomotion parameters for circadian rhythm analysis in fish for the first time.

For the zebrafish research community, the most popular method to conduct circadian rhythm locomotion activity measurement is based on using commercial instruments and software (like ZebraCube from Viewpoint Company, http://www.viewpoint.fr/en/p/equipment/zebracube). However, the cost of such instruments and third-party software is usually unaffordable to most researchers. This disadvantage encouraged us to assemble a low-cost instrument coupled with the two open-source software, idTracker and ImageJ, to perform circadian rhythm studies in fish. The total cost for the entire setup is estimated to be under 3000 USD, while the commercial instrument usually costs around 30,000 USD. In addition, due to growing interest within the zebrafish community, the ImageJ-based method for zebrafish behavioral analyses is gaining popularity in this field. However, most of these studies were conducted mostly in zebrafish larvae, while studies in adult zebrafish are quite rare (Nema et al., 2016). Moreover, most of these methods have been focused on other zebrafish behaviors, such as avoidance behavior (Pelkowski et al., 2011), anxiety-related behavior (Richendrfer et al., 2012) and locomotion behavior (Colwill and Creton, 2011). Previous circadian rhythm-related studies have been focused on larvae fish, thus, the circadian rhythm for adult fish is seldom investigated. In zebrafish, the circadian rhythm patterns in larvae and adults are distinct. Larvae show high activity during the dark cycle (scotophase) and sleep-like behavior during the light cycle (photophase) (Padilla et al., 2011). On the other hand, adult zebrafish display sleep-like behavior during the dark cycle and high activity during the light cycle. We believe our simple, cost-effective device can provide a useful platform to screen genetic mutants in adult zebrafish with circadian rhythm deficiency with high resolution and reliability.

## DISCUSSION

### Comparison of performance between idTracker and ImageJ for circadian rhythm measurement

In this report, we demonstrated how two image-based methods, idTracker and ImageJ, can be used to calculate the circadian rhythm locomotion activity for both diurnal (zebrafish) and nocturnal (catfish) fish species. After cross-validation, both methods can provide consistent and reliable data for circadian rhythm locomotion analysis (Fig. 2B–G, Figs S1A–C, S2 and S3). The pros and cons of each method are compared and summarized in Table 1. The most significant advantage for the ImageJ-based method is a faster calculation speed by running a macro script. Although the detailed parameters such as swimming speed, angular velocity and meandering could not be obtained, the ImageJ-based method provides an excellent tool for quick screening and evaluation. For example, it only takes 3 min of analysis time for videos with 18 fish (six tanks with three fish for each tank) using the ImageJ-based method. However, it will take around 13 min to finish data analysis using the idTracker-based method. Therefore, we recommend the ImageJ-based method as an initial screening platform. Once some interesting differences in circadian rhythm pattern were detected using this fast and simple ImageJ-based method, we could then apply the idTracker-based method to extract more detailed locomotion parameters such as swimming speed, angular velocity and meandering. However, one must keep in mind before using this ImageJ-based method that this method is very sensitive towards background noise, such as dark-line artefacts induced by camera-acquisition speed with respect to the LED lights. Therefore, noise removal is a prerequisite before conducting this experiment, as noise can disrupt the analysis, as mentioned in Table 1.

**Table 1.**
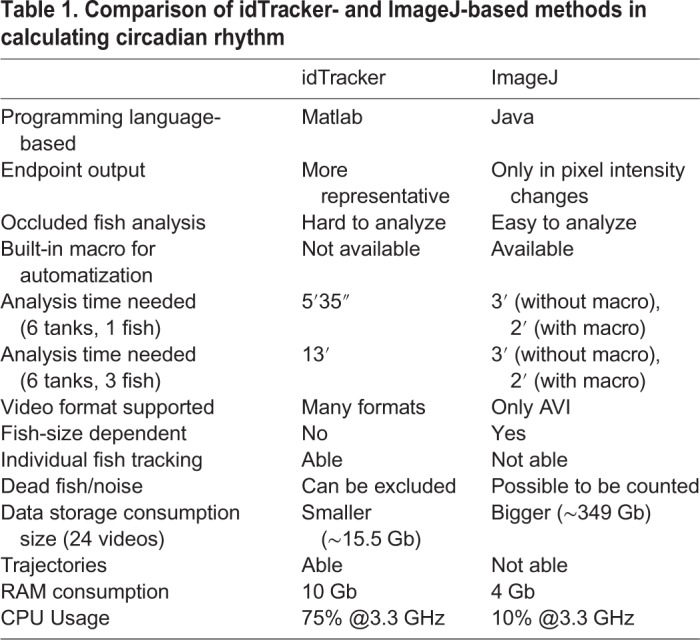
**Comparison of idTracker- and ImageJ-based methods in calculating circadian rhythm**

In conclusion, we have established a cost-effective and easy setup device for circadian rhythm analysis, and also validated two algorithms (idTracker and ImageJ) suitable to perform circadian rhythm locomotion measurement for both diurnal (zebrafish) and nocturnal (catfish) fish species for the first time. The method reported here can be used to evaluate the potential toxicity of chemicals on fish, perform psychological drug screenings on regulating the circadian rhythm and screen genetic mutations displaying circadian rhythm impairment.

## MATERIALS AND METHODS

### Animal ethics

All the experimental protocols and procedures involving zebrafish were approved by the Committee for Animal Experimentation of the Chung Yuan Christian University (Number: CYCU104024, issue date 21 December 2015). All experiments were performed in accordance with the guidelines for laboratory animals. Zebrafish (AB strain) were obtained from Taiwan Zebrafish Core Facility at Academia Sinica, Taipei, Taiwan, and catfish (*C. batrachus*) were purchased from a local aquarium. Catfish were transferred to the Zebrafish Core Facility and acclimatized to the core facility photo regime for ∼1 month. The Zebrafish Core Facility maintained a 12:12 photo regime. Acclimation was necessary to habituate both of the fish in the current photo period condition with white-color LED light as substitute for natural light during the light cycle. Natural daylight has color temperatures ranging from 6000 K under overcast conditions to as high as 20,000 K under clear blue sky conditions during midday periods. However, in the evening hours, the color temperature of the sun decreases to only 2000 K. Meanwhile, common LED lights have stable color temperatures ranging from 4000 K–4500 K (Schubert and Kim, 2005).

### Zebrafish exposed to EtOH

For the acute toxicity test, adult zebrafish aged 6 months were exposed to EtOH for either 30 min (acute) or 7 days (chronic) at a low dosage of 0.1% concentration. About 90% of the water was changed every 24 h with redosing after each change. After EtOH exposure, fish were moved into the circadian rhythm measuring chamber for filming and locomotion tracking.

### Circadian rhythm chamber construction

Sleep–wake behaviors were evaluated using a custom-designed circadian rhythm chamber (designed by Zgenebio Inc., http://www.zgenebio.com/). The light–dark cycle test apparatus consisted of six small plastic fish tanks (20×10 cm) which were placed above a custom-made light box containing IR LEDs and white-color LED stripes arrayed side-by-side. For the light cycle, white-color LEDs were turned on, triggered by timer 1. For the dark cycle, IR LEDs were turned on, triggered by timer 2. An IR-sensitive CCD camera with magnifying lens without image distortion was located above the experimental setup to record the fish movements at 30 frames per second (fps). To maintain video frame rate, the IR CCD camera was connected to a workstation computer with a 1 TB SSD (PLEXTOR M9PeY PCIe, Taipei, Taiwan) and advanced CPU in order to process the data writing and reading efficiently. In addition, Total Recorder software (http://www.totalrecorder.com) was used to perform schedule recording. The whole assembly was placed inside a thermal incubator and the regular temperature for circadian rhythm testing was set at 25°C.

### Video tracking and data analysis

The locomotion activity in the videos was analyzed by using open-source software. For the idTracker-based method (Audira et al., 2018; Pérez-Escudero et al., 2014), the X and Y coordinates of the tracked animals were extracted from the videos frame-by-frame and analyzed following the method described in the Supplementary information (see protocol). For the ImageJ-based method (Abràmoff et al., 2004), the dynamic pixel-change method was employed for locomotion activity extraction. The detailed protocol for data analysis can be found in the Supplementary information (see protocol). All tests were analyzed by either the parametric test (unpaired *t*-test or Dunnett's multiple comparisons test) or the non-parametric test (Mann–Whitney test) based on the data group for each experiment. Due to the differences between the idTracker and ImageJ result unit, data normalization is required in order to compare the light and dark cycle data patterns. Data normalization was done using GraphPad Prism software – with 0% defined as the smallest value in each data set and 100% defined as largest value in each data set – and analyzed with an unpaired *t*-test with Welch's correction, since we cannot assume that these two populations have equal standard deviations. Later, a correlation test was also conducted in order to validate the equivalence of the two methods’ results. As every data set consisted of both of light and dark cycles results, Spearman’s test – a test that makes no assumption about the distribution of the values – was used. Using this nonparametric correlation test, the correlation coefficient (*r*) can be obtained. If the value of *r* is between −1 and 1, it means that these two variables tend to negatively or positively associate together, while an *r*-value of 0 means that the two variables do not correlate at all.

### Construction of a cost-effective light box chamber for circadian rhythm study

In this study, we aimed to establish an easy-to-set-up device that consisted of a custom-made light box chamber equipped with white 940 nm LED light strips as light sources suitable for studying circadian rhythm of diurnal (i.e. zebrafish) and nocturnal (i.e. catfish) fish species with reliability and reproducibility. Six small acrylic fish tanks (20×10 cm) were placed above a light box (Fig. 1A). The light box (48×48 cm) contained 12 strips of white chip on board (COB) LEDs (each strip contained 15 COB LED bubbles) as the light source in the light cycle (Fig. 1B), and 10 strips of 940 nm IR LEDs (each strip contained 15 LED bubbles) as the light source in the dark cycle (Fig. 1C). They were arranged side-by-side as shown in Fig. 1D. The timers, which connected between the light box and power source, were used to control the timing for the light cycle (day cycle, COB LED on) and dark cycle (night cycle, 940 nm IR LED on). To reduce video distortion, a 940 nm IR CCD camera with a magnifying lens was used to record fish movements at 30 fps. Using this device, we were able to track the zebrafish locomotion during the light and dark cycles with high-resolution output video (up to 1028×1024 pixels) (Fig. 1B,C). The light/dark cycle was set as 12:12 photo-regimen. Next, we recorded the fish locomotion activity for 1 min at 1 h intervals and then used either idTracker (Pérez-Escudero et al., 2014) or ImageJ (Abràmoff et al., 2004) software to analyze locomotion activities. The video format was converted from compressed AVI to uncompressed AVI by VirtualDub software (https://sourceforge.net/projects/virtualdub/) in order to be compatible with ImageJ software (Nema et al., 2016). Such video format conversion is unnecessary for idTracker software. Finally, the relative locomotion activities obtained from both algorithms were compared using statistical software GraphPad Prism.

### Measurement of the locomotion activity of fish using idTracker or ImageJ

Here, we provide two simple and easy protocols to perform circadian rhythm activity measurement. The schematic in Fig. 2A summarizes the entire analysis pipeline. The first is an idTracker-based method that can be used to track locomotion at single-individual resolution. The second protocol is an ImageJ-based method that can be used to measure fish locomotion in a quick and simple manner by using macro-language script (detailed protocols for both methods are attached in the Supplementary information under Supplementary protocol). For the idTracker-based protocol, the X and Y coordinates can be extracted from videos for each individual, making it possible to measure the fish locomotion activity more precisely. idTracker is also able to obtain some important parameters, such as swimming speed, swimming distance, turning angle, angular velocity, meandering, freezing, swimming and rapid time–movement ratio. After every parameter for each time interval obtained, we grouped the parameters by their cycle and conducted statistical analysis. Furthermore, for the ImageJ-based protocol, the dynamic pixel changes of adjacent images were extracted frame-by-frame, making it possible to qualitatively measure the overall fish locomotion activity in a more robust manner with the aid of macro language. However, the quantitative information such as swimming speed, swimming distance, turning angle, angular velocity, meandering, freezing, swimming and rapid time–movement ratio are unobtainable when using the ImageJ-based protocol.

## Supplementary Material

Supplementary information
