# Supplementary material for: Establishing simple image-based methods and a cost-effective instrument for toxicity assessment on circadian rhythm dysregulation in fish
Source: Biol Open. 2019 Jun 10;8(6):bio041871. doi: 10.1242/bio.041871 (PMC6602318; doi:10.1242/bio.041871)
Supplement: Supplementary information [file biolopen-8-041871-s1.pdf]

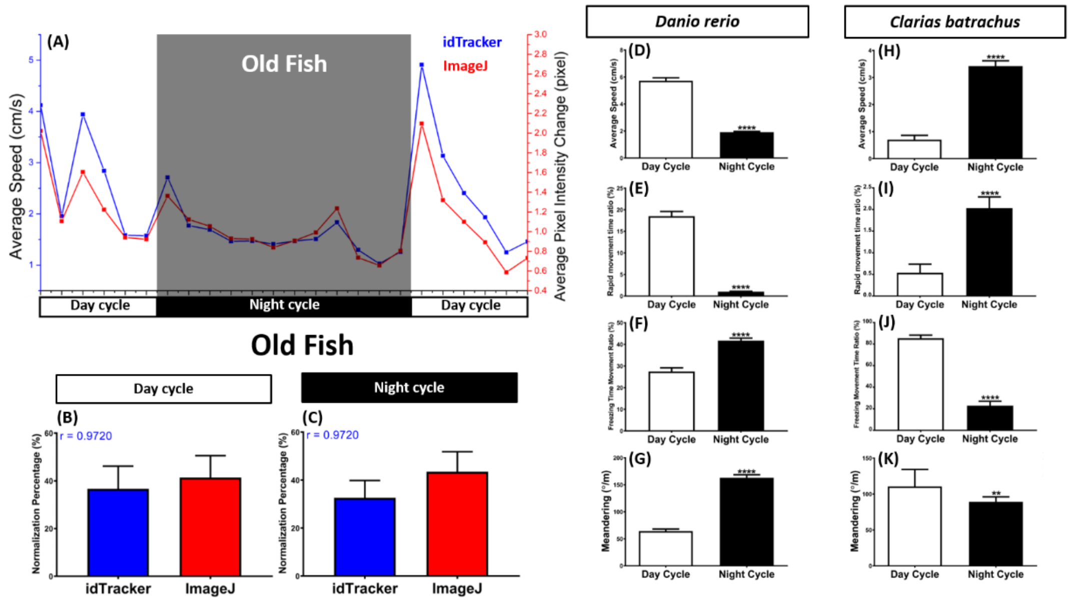

Figure S1. (A) Comparison of 16 months old fish zebrafish circadian rhythm pattern; the locomotion was analyzed by either idTracker (blue color) or ImageJ (red color) methods. The data are expressed as the means. Comparison of idTracker and ImageJ results after data normalization for 16 months old fish in (B) light and (C) dark cycle with r value from Spearman nonparametric correlation test. The data are expressed as the means ± SEM and were analyzed by unpaired *t-test* with Welch's correction (n =18). Comparison of total (D) average speed, (E) average angular velocity, and (F) meandering of zebrafish between the light and dark cycles. The data are expressed as the means ± SEM (n=17) and were analyzed by unpaired *t-test*. Comparison of total (G) average speed, (H) average angular velocity, and (I) meandering of catfish between the light and dark cycles. The data are expressed as the means ± SEM (n=6) and were analyzed by Mann-Whitney test. Significance difference was defined as **P < 0.01, ****P < 0.0001.

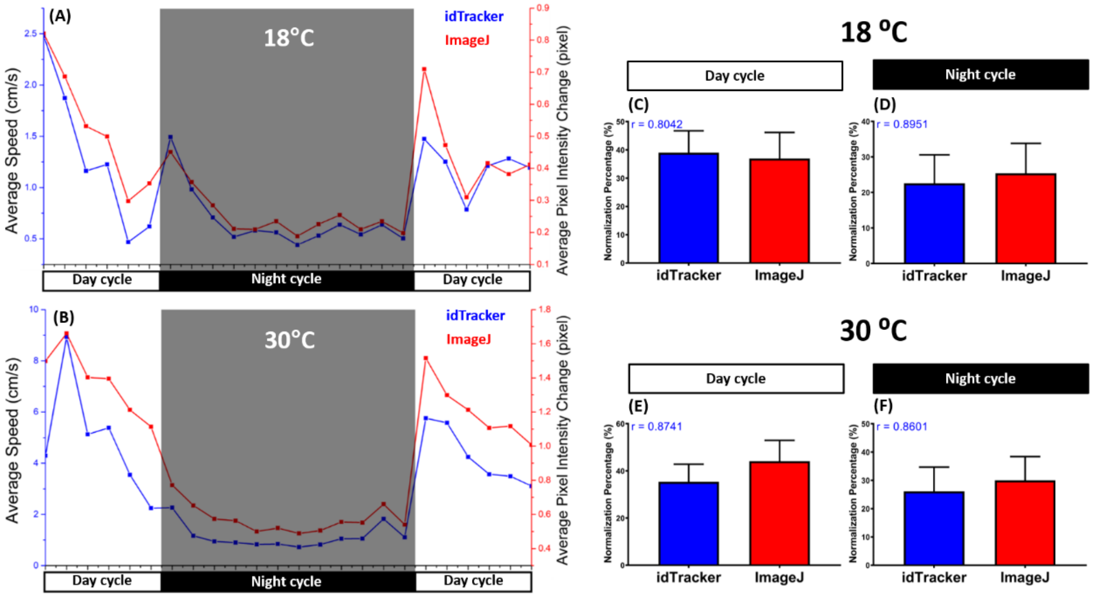

Figure S2. Comparison of zebrafish circadian rhythm activities for fish acclimated to (A) 18°C or (B) 30°C temperature and the locomotion was analyzed by either idTracker (blue color) or ImageJ (red color). The data are expressed as the means. Comparison of idTracker and ImageJ results after data normalization for 18°C acclimated fish in (C) light and (D) dark cycle and 30°C acclimated fish in (E) light and (F) dark cycle with r value (highlighted in blue color) from Spearman nonparametric correlation test. The data are expressed as the means ± SEM and were analyzed by unpaired *t-test* with Welch's correction (n =13 for 18°C temperature group and n=18 for 30°C temperature group).

Biology Open • Supplementary information

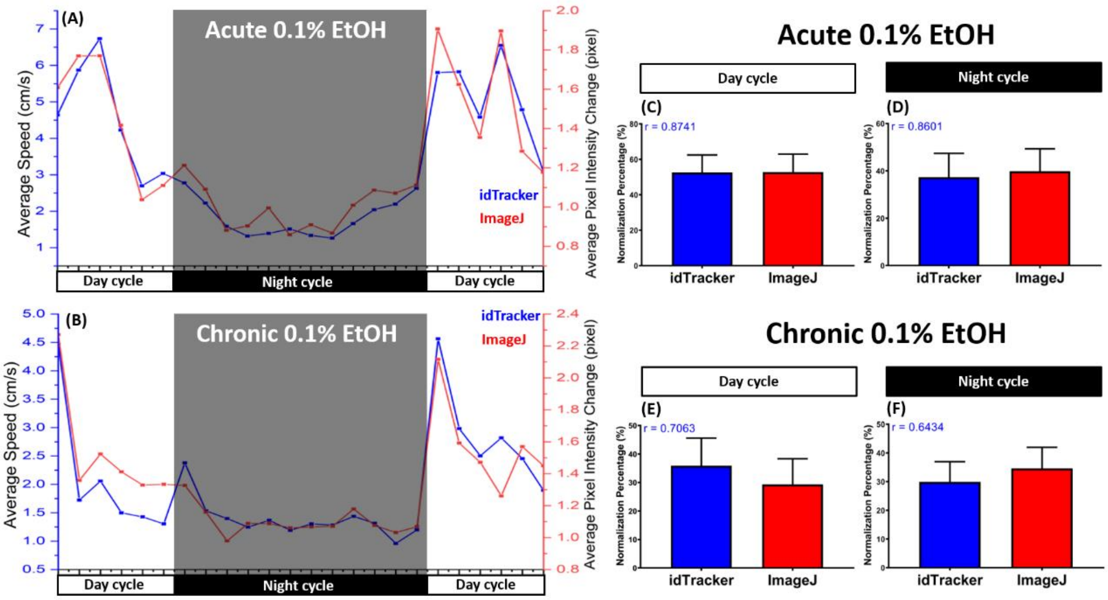

Figure S3. Comparison of zebrafish circadian rhythm activities for fish acutely (A) andchronically (B) exposed to 0.1% EtOH and the locomotion was analyzed by either idTracker (blue color) or ImageJ (red color). The data are expressed as the means. Comparison of idTracker and ImageJ results after data normalization for acutely 0.1% EtOH exposed fish in light (C) and dark (D) cycle and chronically 0.1% EtOH exposed fish in light (E) and dark (F) cycle with r value (highlighted in blue color) from Spearman nonparametric correlation test. The data are expressed as the means ± SEM and were analyzed by unpaired *t-test* with Welch's correction (n =18 for acute and chronic exposure of 0.1% EtOH groups).

Biology Open • Supplementary information

Table S1

References	Age of Zebrafish	Light Source	Recording Apparatus	Recording Quality	Data Analysis	Data output
Cahill et al. (1998) [1] & Hurd et al. (2002) [2]	Larvae	Infrared (>700 nm) illumination	CCD camera with a 28 mm lens, automatic gain control and shading correction (Hamamatsu Photonics, Hamamatsu, +F2+D2:H+D2:I2	Monochrome images (640 x 480 pixels, 8 bit resolution)	Optimas (Seattle, WA) image analysis software controlled by a macro written in Analytical Language for Images & Chrono II software (T. Roenneberg, University of Munich)	Actogram plots
Hirayama et al. (2005) [3]	Larvae	A custom made diffuse axial illuminator with infrared light source	Monochrome video camera with a 50-mm macro lens, and a 1.7-cm CCD sensor with the IR-blocking filter removed, automatic gain control, shading correction and horizontal center resolution >750 TVL; a desktop computer with a 640x480 pixel, 8-bit gray scale frame capture card (Flashpoint 128), and a Windows operating system	Not mentioned in the original article	Optimate 6.2 (MediaCybernetics, Silver Spring, MD) image processing software with the Swimming1.1 macro (Meyer Instruments, Houston, TX) and Chrono 4.5.1	Actogram plots
Prober et al. (2006) [4]	Larvae	Zebrabox (ViewPoint Life Sciences)	An automated video-tracking system (Videotrack; ViewPoint Life Sciences, Montreal, Quebec, Canada) with a Dinion one-third inch Monochrome camera (model LTC0385; Bosch, Fairport, NY) fitted with a fixed-angle megapixel lens (M5018-MP; Computar) and infrared filter	Not mentioned in the original article	Custom PERL software and Visual Basic Macros for Microsoft (Seattle, WA) Excel	Activity plots and 4 behavioral endpoints

Biology Open • Supplementary information

Zhdanova et al. (2008) [5]	Adult Male	Illuminated floor and an additional light source placed next to the tanks	One camera & automatic animal tracking software (Video-track, View Point Inc, France)	30 fps video	Nonlinear least squares analysis (Mathematica, Wolfram Research, Champaign, IL)	4 behavioral endpoints
Lopez-Olmeda et al. (2009) [6]	Adult	Fluorescent bulb (F15W/GRO. Sylvania Gro-Lux, Germany) with 400 lux light intensity	Infrared photocell (Omron, mod E3S-AD62, Kyoto, Japan	No video (light-beam interruptions)	Chronobiology software *El Temps*© (version 1.228; Prof. Diez-Noguera, University of Barcelona) and SPSS® software	Actogram plots
This Study (2018)	Adult	White COB LED and 940 nm IR LED beneath the fish tanks	940 nm infrared CCD camera, workstation computer with 1 TB SSD and Intel Core i7-6850K CPU, and Total Recorder software	1028x1024 30 fps video	Image-J image analysis software, idTracker software, and Microsoft Excel	Average speed and meandering plots and 6 behavioral endpoints

REFERENCES

1. Cahill, G.M., M.W. Hurd, and M.M. Batchelor, *Circadian rhythmicity in the locomotor activity of larval zebrafish.* Neuroreport, 1998. **9**(15): p. 3445-3449.
2. Hurd, M.W. and G.M. Cahill, *Entraining signals initiate behavioral circadian rhythmicity in larval zebrafish.* Journal of biological rhythms, 2002. **17**(4): p. 307-314.
3. Hirayama, J., et al., *Analysis of circadian rhythms in zebrafish*, in *Methods in enzymology*. 2005, Elsevier. p. 186-204.
4. Prober, D.A., et al., *Hypocretin/orexin overexpression induces an insomnia-like phenotype in zebrafish.* Journal of Neuroscience, 2006. **26**(51): p. 13400-13410.
5. Zhdanova, I., et al., *Aging of the circadian system in zebrafish and the effects of melatonin on sleep and cognitive performance.* Brain research bulletin, 2008. **75**(2-4): p. 433-441.
6. López-Olmeda, J.F. and F.J. Sánchez-Vázquez, *Zebrafish temperature selection and synchronization of locomotor activity circadian rhythm to ahemeral cycles of light and temperature.* Chronobiology international, 2009. **26**(2): p. 200-218.

Supplementary protocol

Method 1 : Establish idTracker-based method to measure circadian rhythm in fish

Required Programs summarized

- Matlab Compiler Runtime

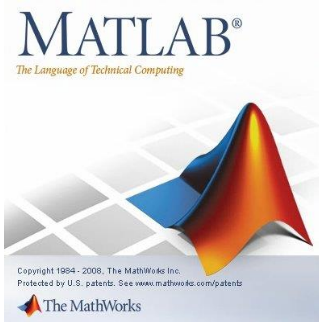

- idTracker

- ImageJ

- Total Recorder

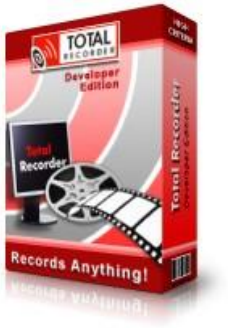

- Microsoft Excel

Methods

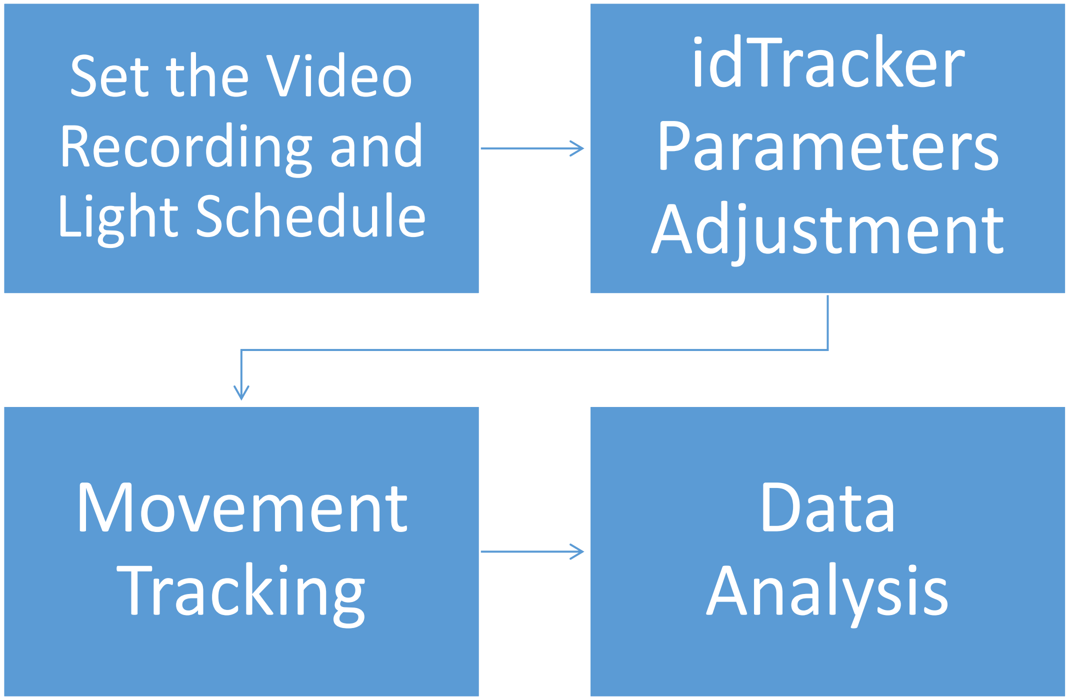

Biology Open • Supplementary information

Tips for Video Recording

- Frame rate per second (FPS)
  - **↑ : more accurate tracking, slower process**
  - ↓ : less accurate tracking, faster process
- Use a good contrast vessel for the fishes
- Using Black and White image setting is recommended because it can enhance the contrast
- There must be free space in the disk because tracker will generate a large amount of data
- Internal hard drive's video usage is strongly recommended

Example Video (Zebrafish)

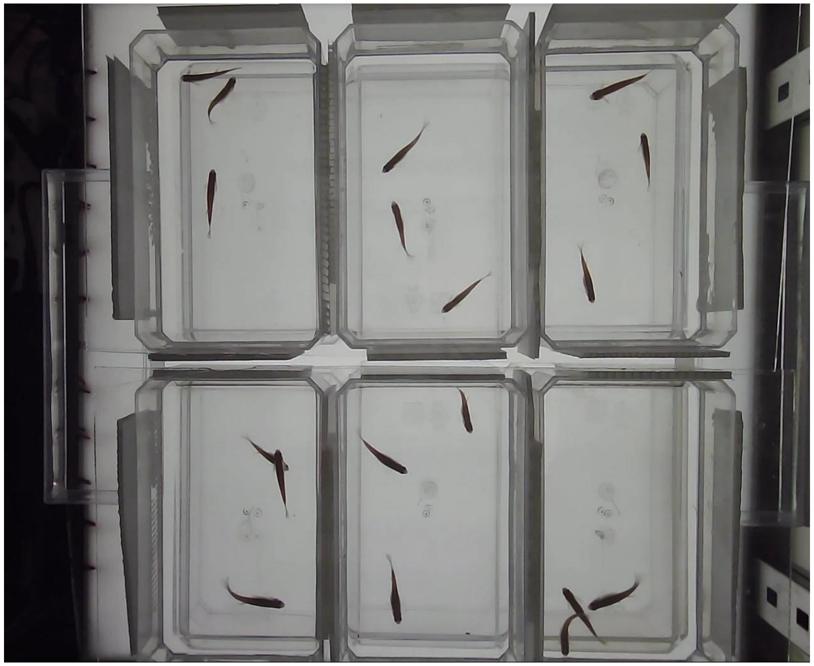

Day Cycle

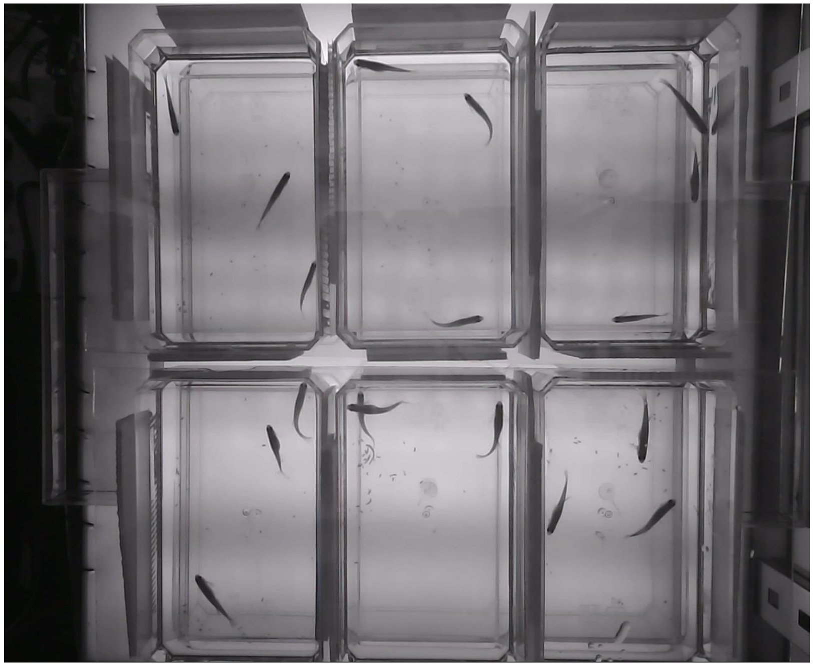

Night Cycle

1280x1024; 30fps

*original video is available upon request

Parameters Adjustments

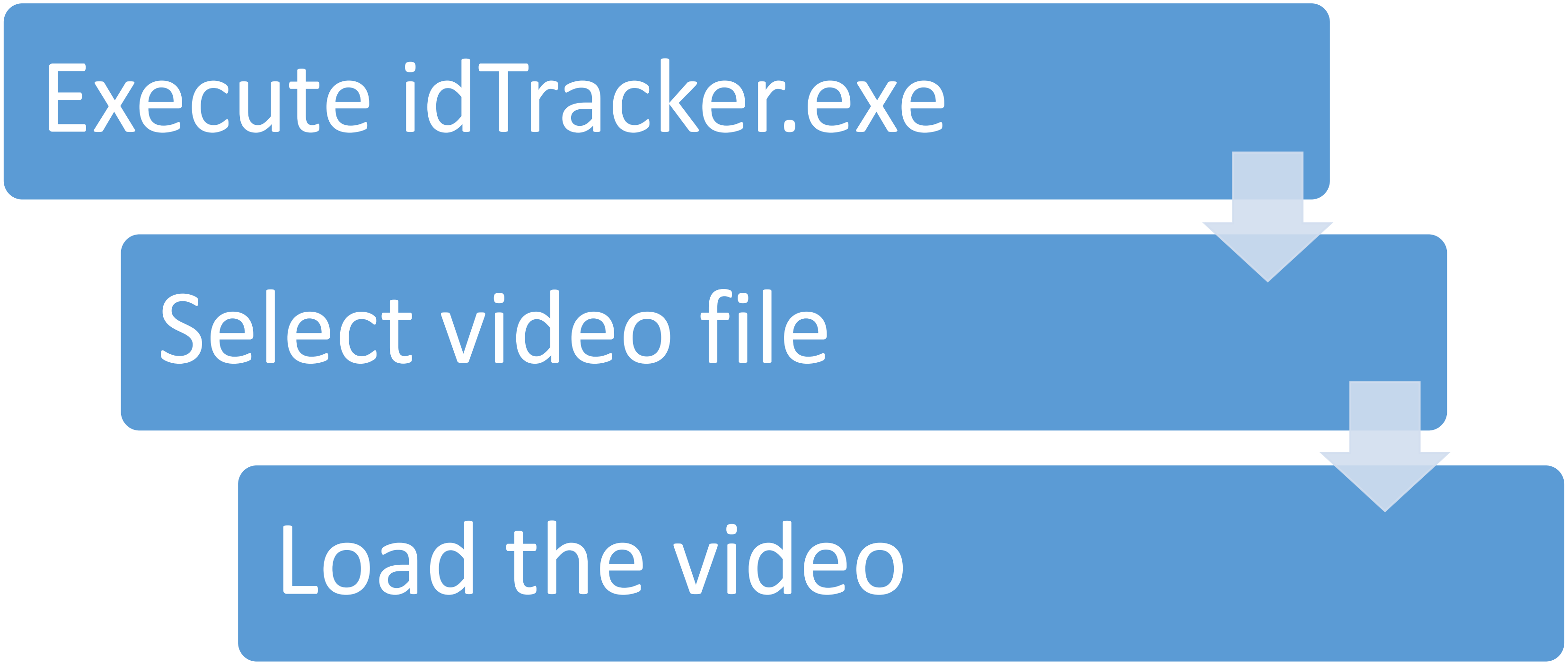

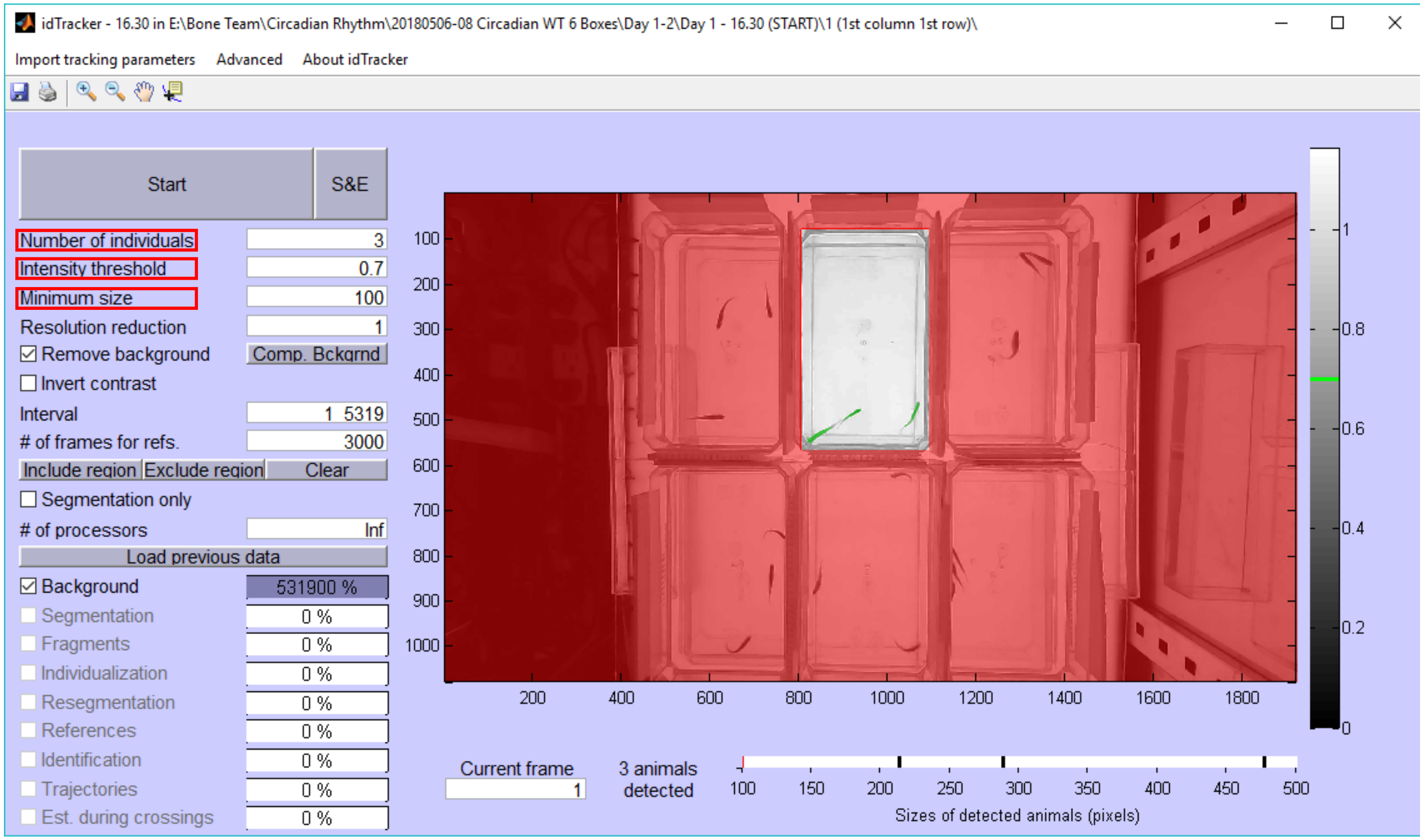

1. Enter the number of individuals

2. Intensity threshold
   - The system is going to considers that pixels with lower intensity than this threshold belong to the animals (*vice versa* if "invert contrast" is checked)

3. Minimum size
   - The programs will reject blobs smaller than the minimum size entered

Biology Open • Supplementary information

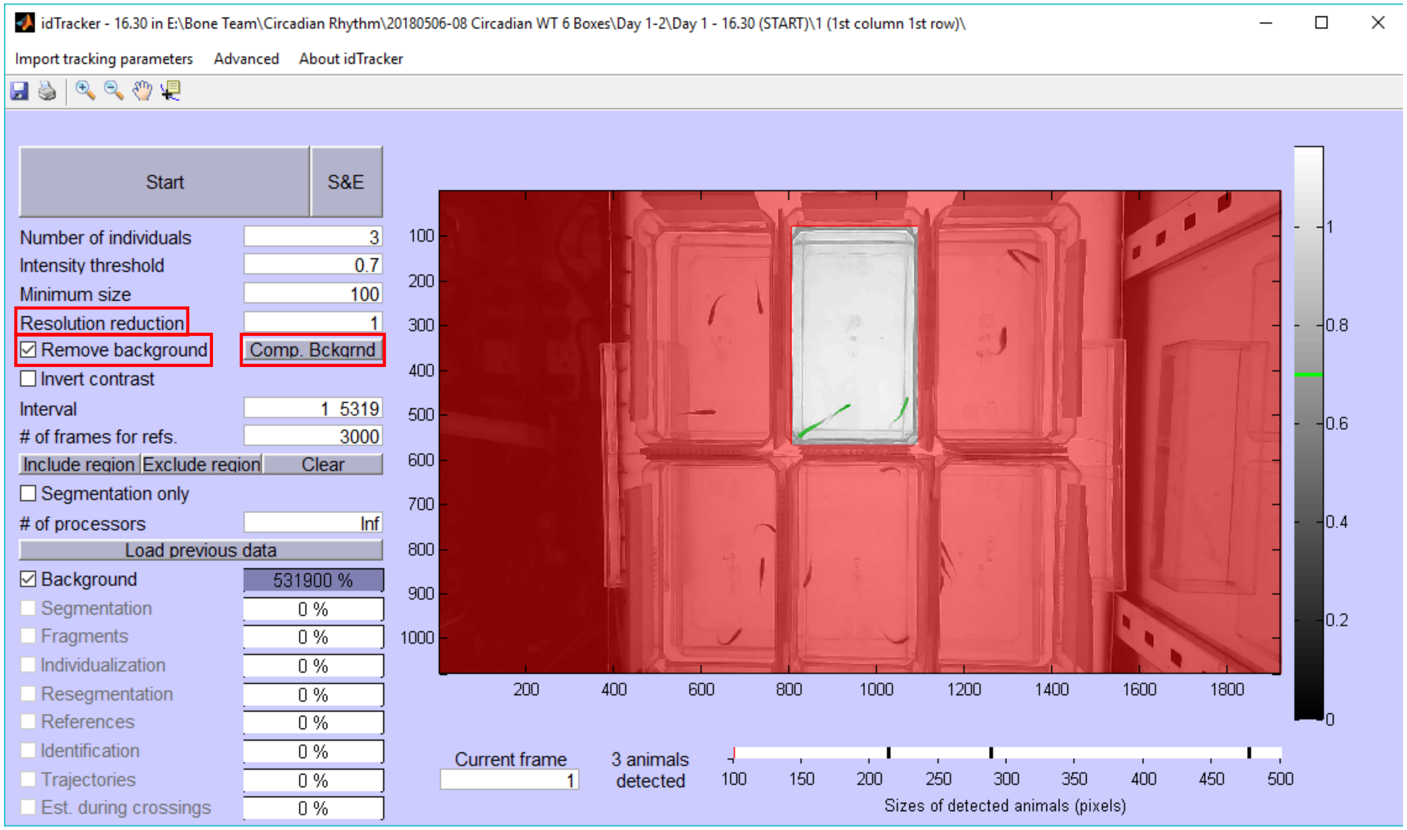

4. Choose resolution reduction

- If the sizes of the animals are bigger than 2000 pixels, input a number higher than 1 (the number of pixels will be divided by n^2, where n is the number in the box)

5. Background removal option

- Check if you want to activate the background removal option
- To compute it before, click on the 'Compute Bckgrnd' button

Biology Open • Supplementary information

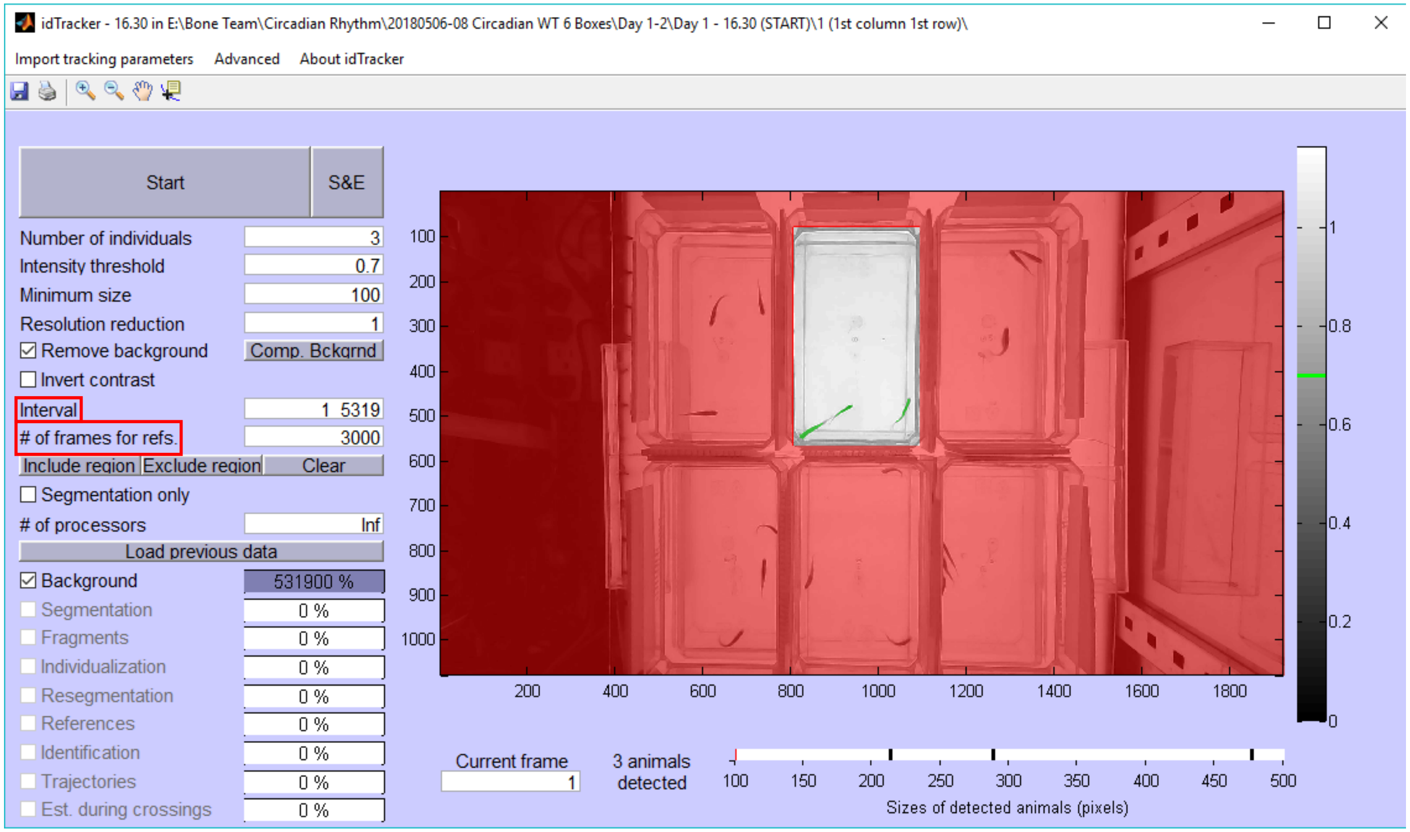

6. Choose an interval

- If you want to track only part of video, enter the interval that you want to track

7. Number of references frames

- Choose a lower number for increased speed, a higher number for increased accuracy

Biology Open • Supplementary information

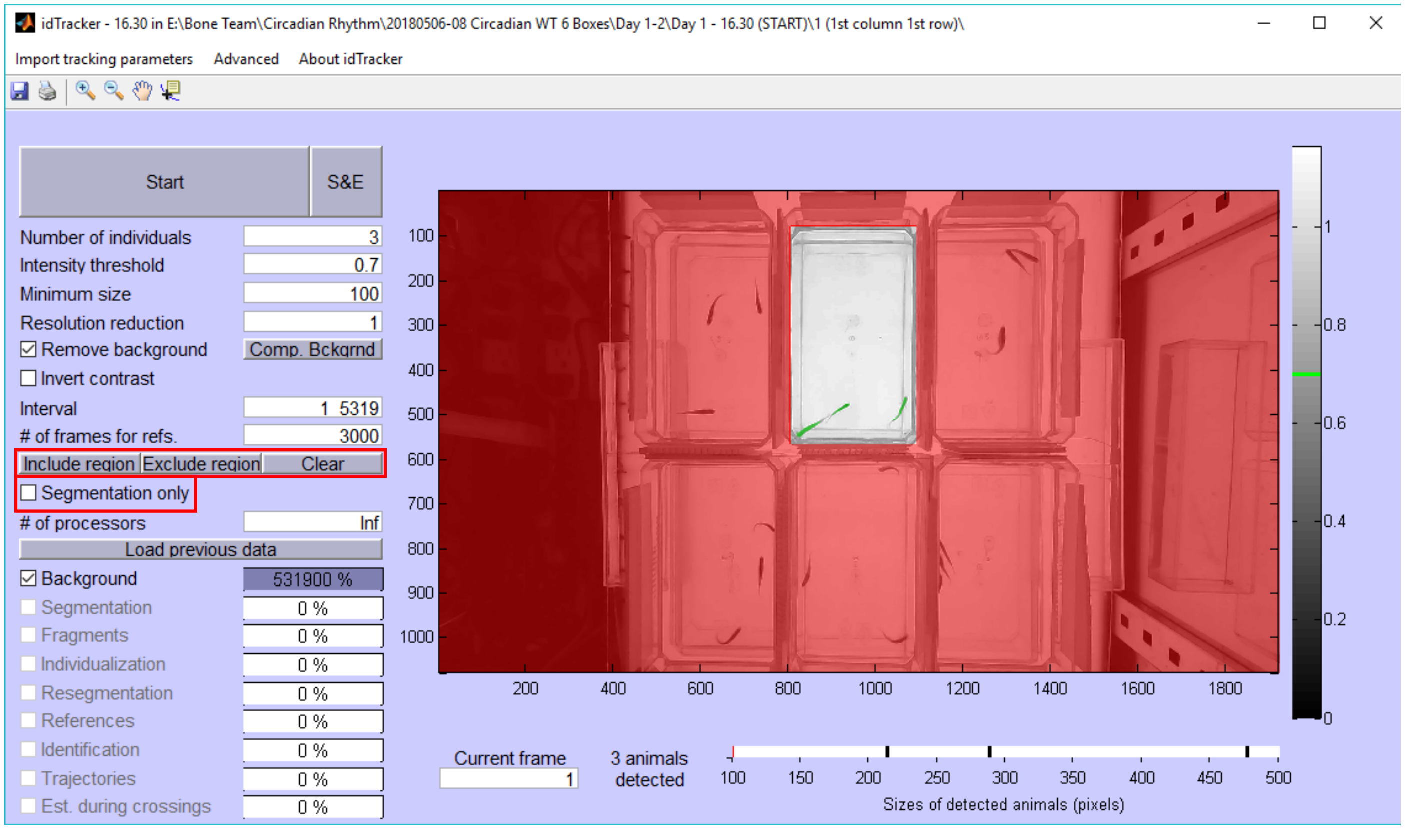

8. Select Region Of Interest (ROI) and/or exclude regions

- Click on button ‘Clear’ to clear all previously defined ROS’s or excluded regions

9. Segmentation only

- If this box is checked, idTracker will exit after the segmentation step, leaving the tracking unfinished.
- The data can be recovered later using button ‘Load Previous Data’

Biology Open (2019): doi:10.1242/bio.041871: Supplementary information

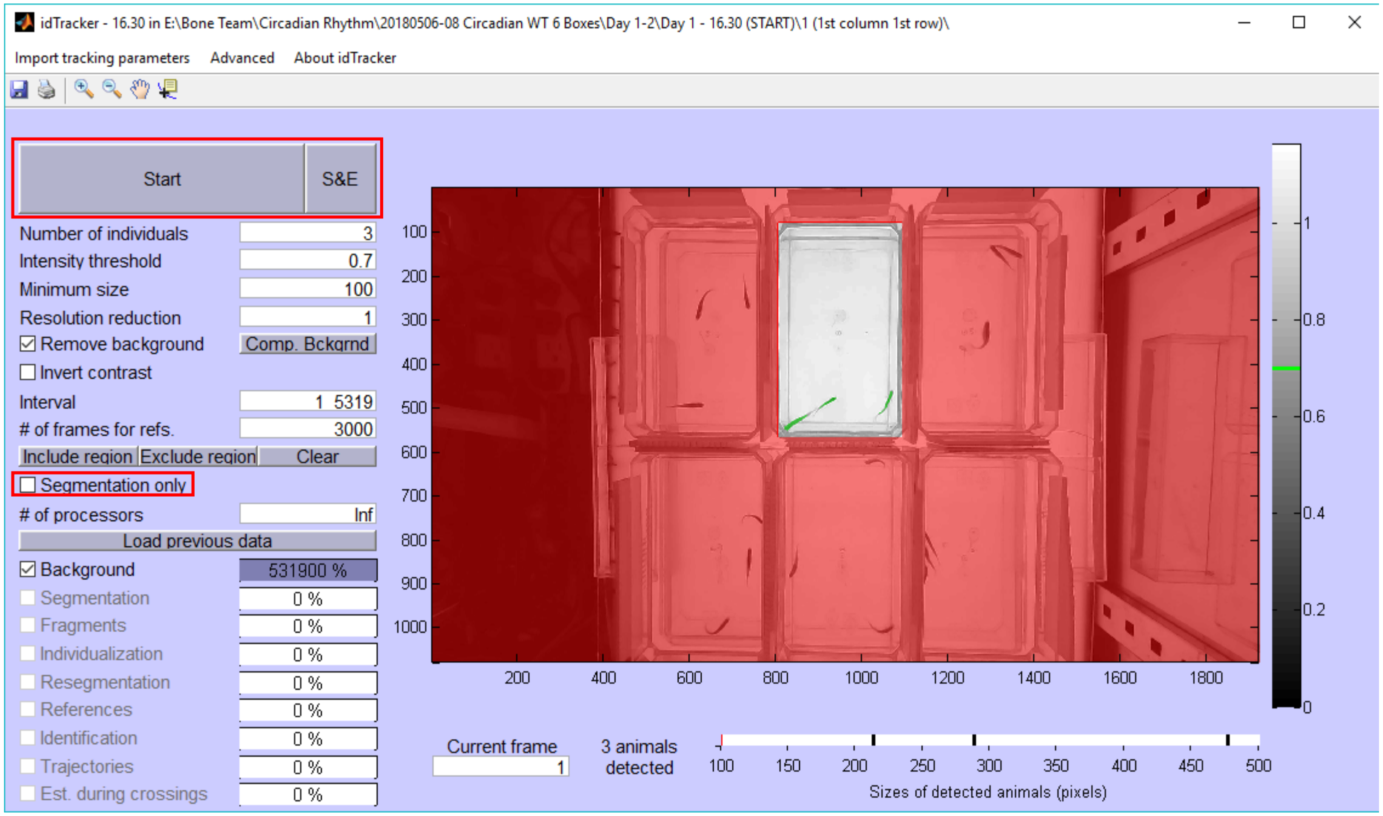

10. Number of processors

- The number of this box indicates how many processors idTracker will use ('Inf' means that idTracker will use all available processors)

11. Start tracking

- Click the 'Start' button
- If 'S&E' (Save & Exit) button pressed, the programs ends, but the tracking does not start. All tracking parameters can be used later using 'Load Previous Data' button

Biology Open • Supplementary information

Movement Tracking

√ Separated tracking X

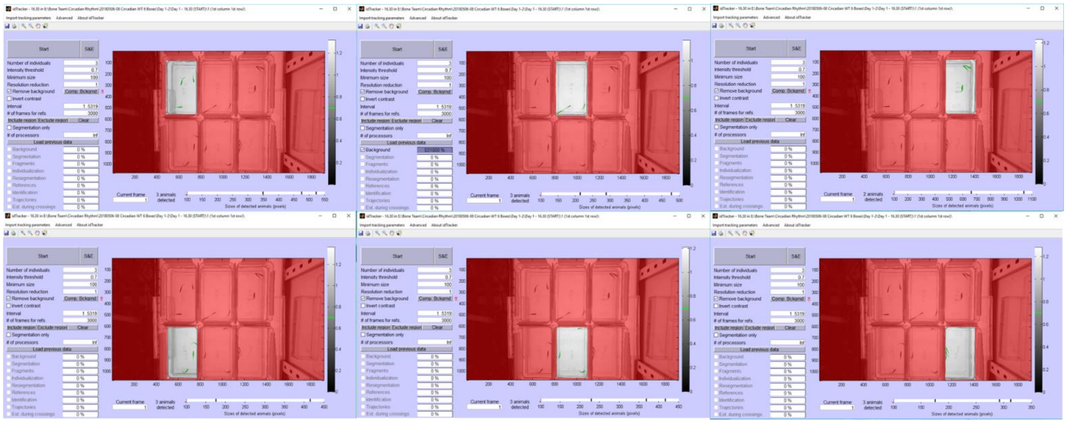

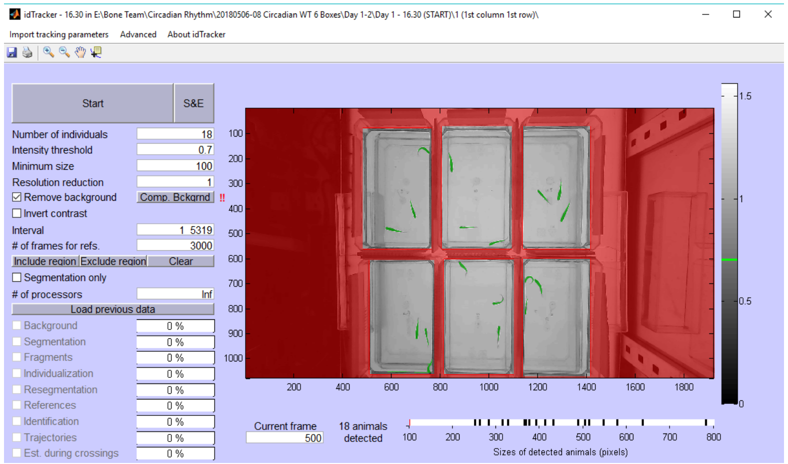

- Advantages
  - **Reduce** error occurrence
  - Tracking **duration** takes **less** time
- Disadvantages
  - Need **more space** to store the video

- Advantages
  - Just **one** video needs to be saved
- Disadvantages
  - Error **often** occurred
  - Tracking **duration** takes **more** time

Biology Open • Supplementary information

Results

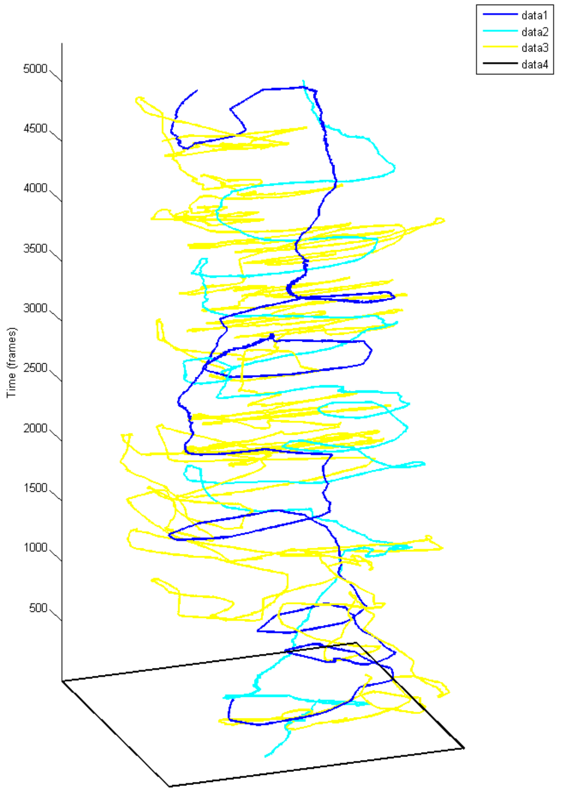

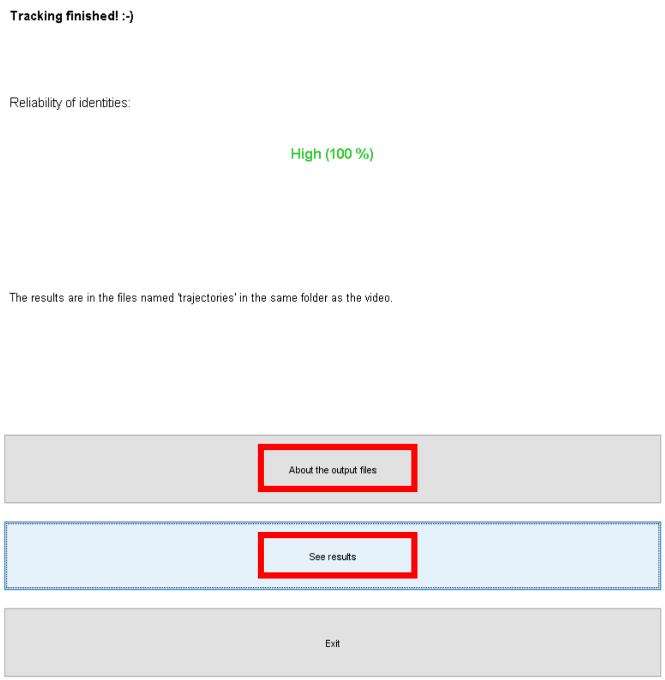

- The results are in a folder called ‘segm’ located in the same folder as the video
- Press ‘About the output files’ for more information about the results
- ‘See results’ button will play the result’s video

Biology Open • Supplementary information

Biology Open (2019): doi:10.1242/bio.041871: Supplementary information

Tracking Result's Video

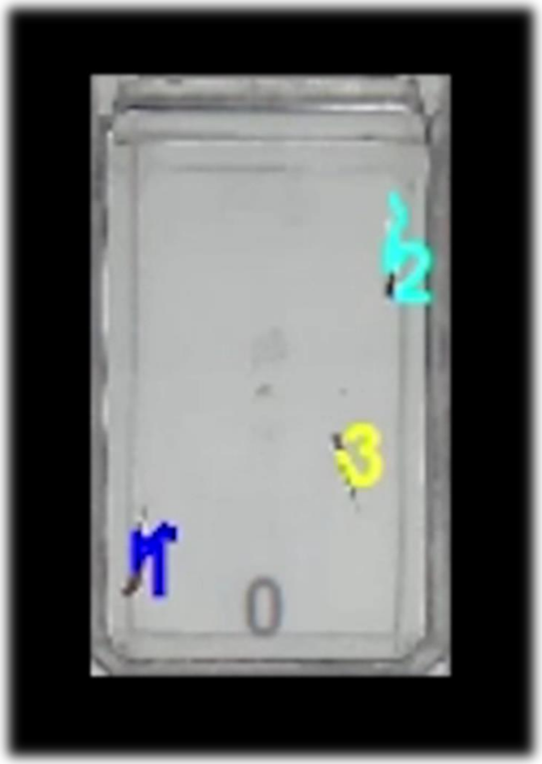

Biology Open • Supplementary information

Results

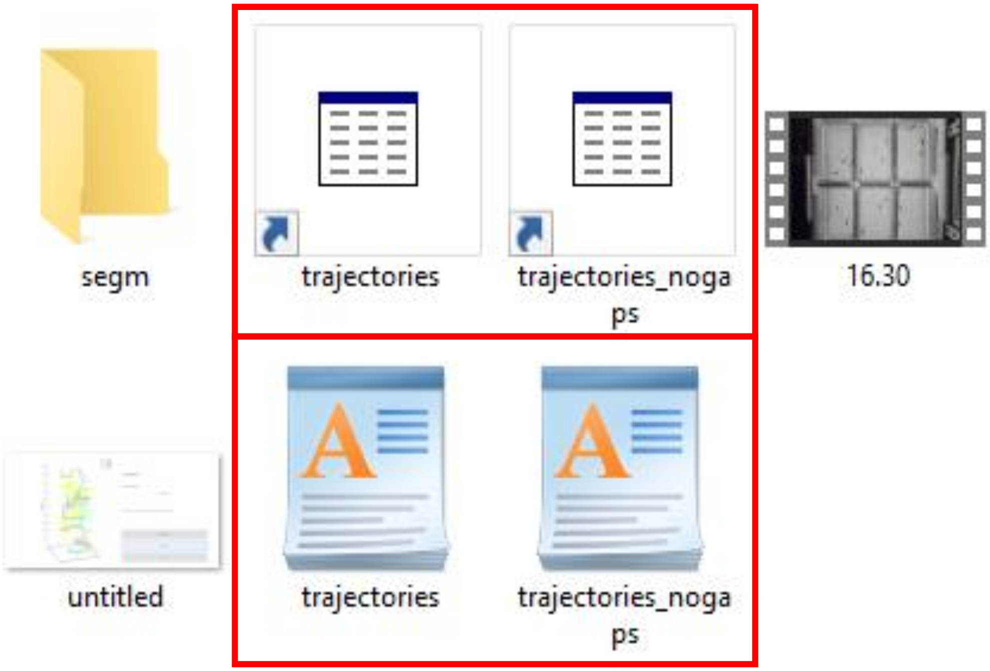

- There will be four output files
  - Two of them are .mat files, to be loaded into Matlab
  - The other two are .txt files to import the data into any software
- Both of the files contain identical information, which is the following
  - X and Y coordinates of each individual in each frame
  - Probability of correct assignment

Results

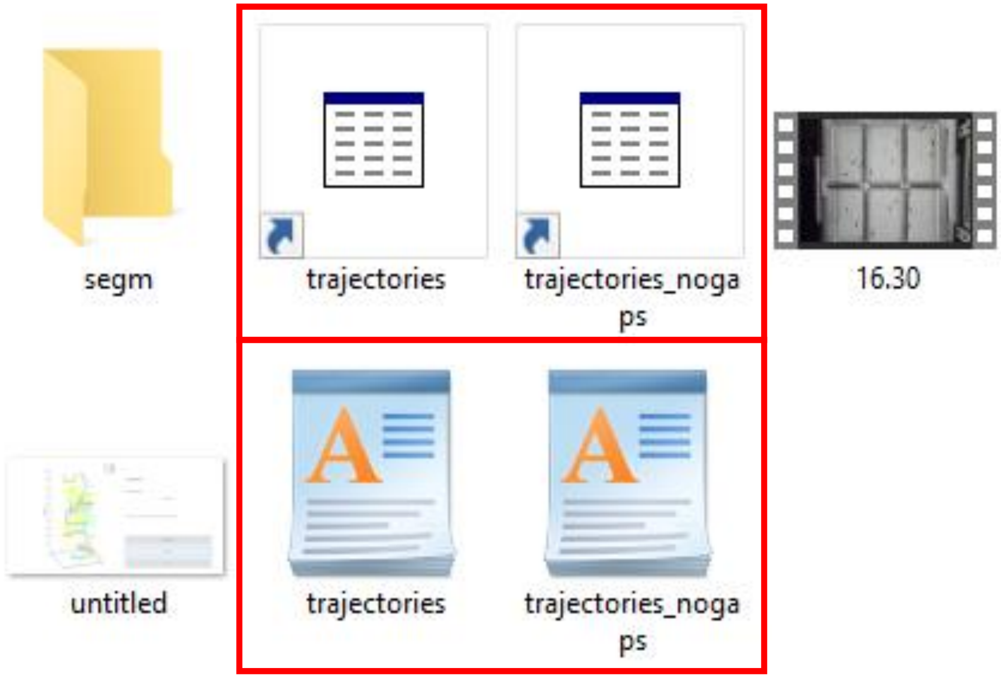

- Difference between trajectories and trajectories_nogaps
  - The files called ‘trajectories’ contain only the position of each individual when it is not occluded
  - The files called ‘trajectories_nogaps’ contain the position of each individual also when occluded
    - The probability of correct identity contains a negative number when the position comes from an estimation

Biology Open (2019): doi:10.1242/bio.041871: Supplementary information

Results

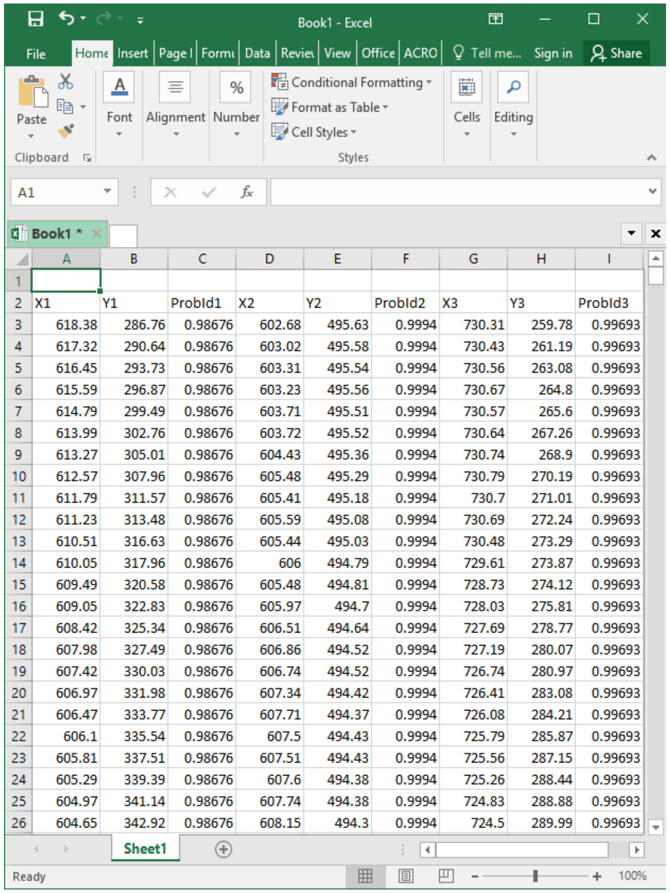

	A	B	C	D	E	F	G	H	I
1									
2	X1	Y1	ProbId1	X2	Y2	ProbId2	X3	Y3	ProbId3
3	618.38	286.76	0.98676	602.68	495.63	0.9994	730.31	259.78	0.99693
4	617.32	290.64	0.98676	603.02	495.58	0.9994	730.43	261.19	0.99693
5	616.45	293.73	0.98676	603.31	495.54	0.9994	730.56	263.08	0.99693
6	615.59	296.87	0.98676	603.23	495.56	0.9994	730.67	264.8	0.99693
7	614.79	299.49	0.98676	603.71	495.51	0.9994	730.57	265.6	0.99693
8	613.99	302.76	0.98676	603.72	495.52	0.9994	730.64	267.26	0.99693
9	613.27	305.01	0.98676	604.43	495.36	0.9994	730.74	268.9	0.99693
10	612.57	307.96	0.98676	605.48	495.29	0.9994	730.79	270.19	0.99693
11	611.79	311.57	0.98676	605.41	495.18	0.9994	730.7	271.01	0.99693
12	611.23	313.48	0.98676	605.59	495.08	0.9994	730.69	272.24	0.99693
13	610.51	316.63	0.98676	605.44	495.03	0.9994	730.48	273.29	0.99693
14	610.05	317.96	0.98676	606	494.79	0.9994	729.61	273.87	0.99693
15	609.49	320.58	0.98676	605.48	494.81	0.9994	728.73	274.12	0.99693
16	609.05	322.83	0.98676	605.97	494.7	0.9994	728.03	275.81	0.99693
17	608.42	325.34	0.98676	606.51	494.64	0.9994	727.69	278.77	0.99693
18	607.98	327.49	0.98676	606.86	494.52	0.9994	727.19	280.07	0.99693
19	607.42	330.03	0.98676	606.74	494.52	0.9994	726.74	280.97	0.99693
20	606.97	331.98	0.98676	607.34	494.42	0.9994	726.41	283.08	0.99693
21	606.47	333.77	0.98676	607.71	494.37	0.9994	726.08	284.21	0.99693
22	606.1	335.54	0.98676	607.5	494.43	0.9994	725.79	285.87	0.99693
23	605.81	337.51	0.98676	607.51	494.43	0.9994	725.56	287.15	0.99693
24	605.29	339.39	0.98676	607.6	494.38	0.9994	725.26	288.44	0.99693
25	604.97	341.14	0.98676	607.74	494.38	0.9994	724.83	288.88	0.99693
26	604.65	342.92	0.98676	608.15	494.3	0.9994	724.5	289.99	0.99693

- Each row corresponds to one frame of the video
  - Columns 1 and 2 are the x and y coordinates of individual 1, respectively, and so on
- There is an estimation of the probability of correct assignment for each frame and it is usually conservative
  - The probabilities for individual 1 are in column 3, for individual 2 in column 6, and so on

Biology Open • Supplementary information

Results

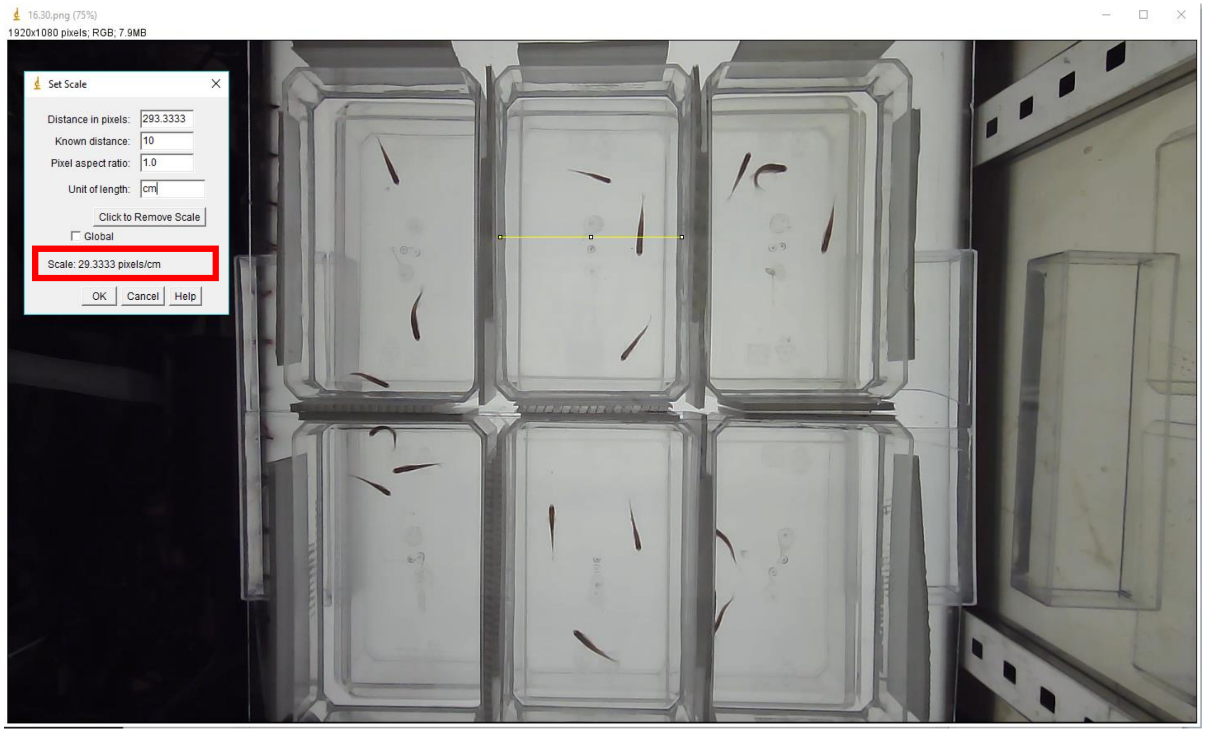

- Convert the pixel dimension into the standard length dimension using **ImageJ**

ImageJ
Image Processing and Analysis in Java

Biology Open • Supplementary information

Biology Open (2019): doi:10.1242/bio.041871: Supplementary information

Results (Calculation Example)

	A	B	C	D	E	F	G	H	I	J	K	L
1			Distance and Velocity						Turning Angle/frame		Average Angular Velocity (°/s)	Meandering (°/m)
2	X1	Y1	Distance (pixel)	Speed (cm/s)	Maximum speed (cm/s)	Minimum speed (cm/s)	Total distance (cm)	Average speed (cm/s)	With Direc	Without D		
3	611.93	165.43	0.012692955	0.380788655	6.936749959	0	49.5680466	0.826593328	-0.03818	0.038181	1.768126286	214.0241233
4	612.28	165.28	0.006009252	0.180277564					0.054786	0.054786		
5	612.22	165.45	0.005962848	0.178885438					0.007984	0.007984		
6	612.14	165.61	0.01982703	0.594810894					0.007215	0.007215		
7	611.87	166.14	0.001943651	0.058309519					-0.0255	0.025498		
8	611.82	166.17	0.005906682	0.177200451					0.065998	0.065998		
9	611.87	166.34	0.004472136	0.134164079					0.007984	0.007984		
10	611.93	166.22	0.027816861	0.834505842					-0.01037	0.010369		
11	611.35	166.82	0.029030635	0.870919055					-0.02155	0.021548		
12	612.07	166.33	0.006262765	0.187882942					0.010796	0.010796		
13	611.99	166.5	0.002981424	0.089442719					0.007984	0.007984		
14	611.95	166.58	0.010203485	0.306104557					-0.00554	0.005543		
15	611.76	166.82	0.015365907	0.460977223					-0.00746	0.00746		
16	611.46	167.17	0.008062258	0.241867732					-0.13883	0.138828		
17	611.7	167.14	0.020002778	0.600083328					0.015327	0.015327		
18	611.46	167.69	0.024413111	0.732393337					-0.0025	0.002498		
19	611.04	168.29	0.009219544	0.276586334					-0.00746	0.00746		
20	610.86	168.5	0.006082763	0.182482876					0.059945	0.059945		
21	610.83	168.68	0.023175658	0.695269732					-0.01535	0.015354		
22	611.36	168.23	0.018601075	0.558032257					-0.00367	0.003666		
23	611.03	168.68	0.019102065	0.573061951					0.003808	0.003808		
24	610.75	169.18	0.006036923	0.181107703					-0.03857	0.038567		
25	610.77	169.36	0.006411795	0.192353841					0.005744	0.005744		
26	610.68	169.53	0.005754226	0.172626765					-0.02462	0.024616		
27	610.71	169.7	0.035087193	1.052615789					-0.00594	0.005943		
28	610.05	170.52	0.022236107	0.667083203					-0.00706	0.007064		
29	610.48	170.01	0.015733898	0.472016949					-0.00378	0.003785		
30	610.2	170.39	0.014974052	0.449221549					-0.10406	0.104056		
31	610.07	170.82	0.01313604	0.39408121					-0.00317	0.003165		
32	609.84	171.14	0.009339284	0.280178515					-0.02089	0.020894		
33	610.07	170.98	0.001666667	0.05					0.018725	0.018725		
34	610.11	171.01	0.004955356	0.148660687					0.008879	0.008879		
35	610.21	171.12	0.020248457	0.607453702					-0.00044	0.000442		
36	609.88	171.63	0.00664162	0.199248588					-0.05339	0.053392		
37	610.07	171.57	0.014636332	0.439089968					0.002752	0.002752		
38	609.85	171.95	0.003901567	0.117046999					0.045839	0.045839		
39	609.96	171.99	0.003887301	0.116619038					-0.0255	0.025498		
40	610.06	171.93	0.011279283	0.338378486					0.073672	0.073672		
41	609.95	172.25	0.009386752	0.281602557					-0.07338	0.073376		
42	609.87	172.52	0.00790218	0.237065392					-0.0302	0.0302		
43	610.08	172.41	0.004055175	0.121655251					-0.1037	0.103696		
44	610.2	172.39	0.016275407	0.488262225					-0.0025	0.002498		

16.30 | 17.30 | 18.30 | 19.30 | 20.30 | 21.30 | 22.30 | 23.30 | 00.30 | 01.30 | 02.30 | 03.30 | 04.30 | 05.30 | ...

- Use the Microsoft Excel's formula to calculate the fish's movement frame by frame
- Measure the fish's rate of speed by dividing the calculation results with the time

Some parameters we can get from this idTracker-based method

- **Total distance traveled (cm)**

$$\Sigma\sqrt{(X_2-X_1)^2+(Y_2-Y_1)^2+(Z_2-Z_1)^2}$$

- **Avg. swimming speed (cm/s)**

$$\frac{Total\ distance\ (cm)}{Total\ Time\ (s)}$$

- **Maximum speed (cm/s)**
- **Minimum speed (cm/s)**
- **Freezing Time (s)**
  (Total Time when speed less than 1cm/s)
- **Swimming Time (s)**
  (Total Time when speed 1-10 cm/s)
- **Rapid Movement Time (s)**
  (Total Time when speed more than 10 cm/s)
- **Meandering (°/m)**
  (The degree of turning vs. travel distance)

$$\left(\frac{Absolute\ turn\ angle\ (^\circ)}{Total\ distance\ (cm)} \times 100\right)$$

Biology Open • Supplementary information

Some parameters we can get from this idTracker-based method

- **Turning Angle (º)**

$$\frac{1}{\frac{tan(\frac{\Delta Y}{\Delta X}) \times 180}{\pi}}$$

- **Fast Type Latent Time (s)**
  (Total time when Angular velocity above 0.5º/ms)

- **Slow Type Latent Time (s)**
  (Total time when Angular velocity below 0.5º/ms)

- **Angular Velocity (º/ms)**

$$Turning\ Angle \times (\frac{Video\ duration\ (ms)}{Frame\ per\ second})$$

- **Absolute Turn Angle**
  (Sum of all the Turning Angles without considering the sign of angular direction)

Biology Open • Supplementary information

Example of circadian rhythm pattern for zebrafish

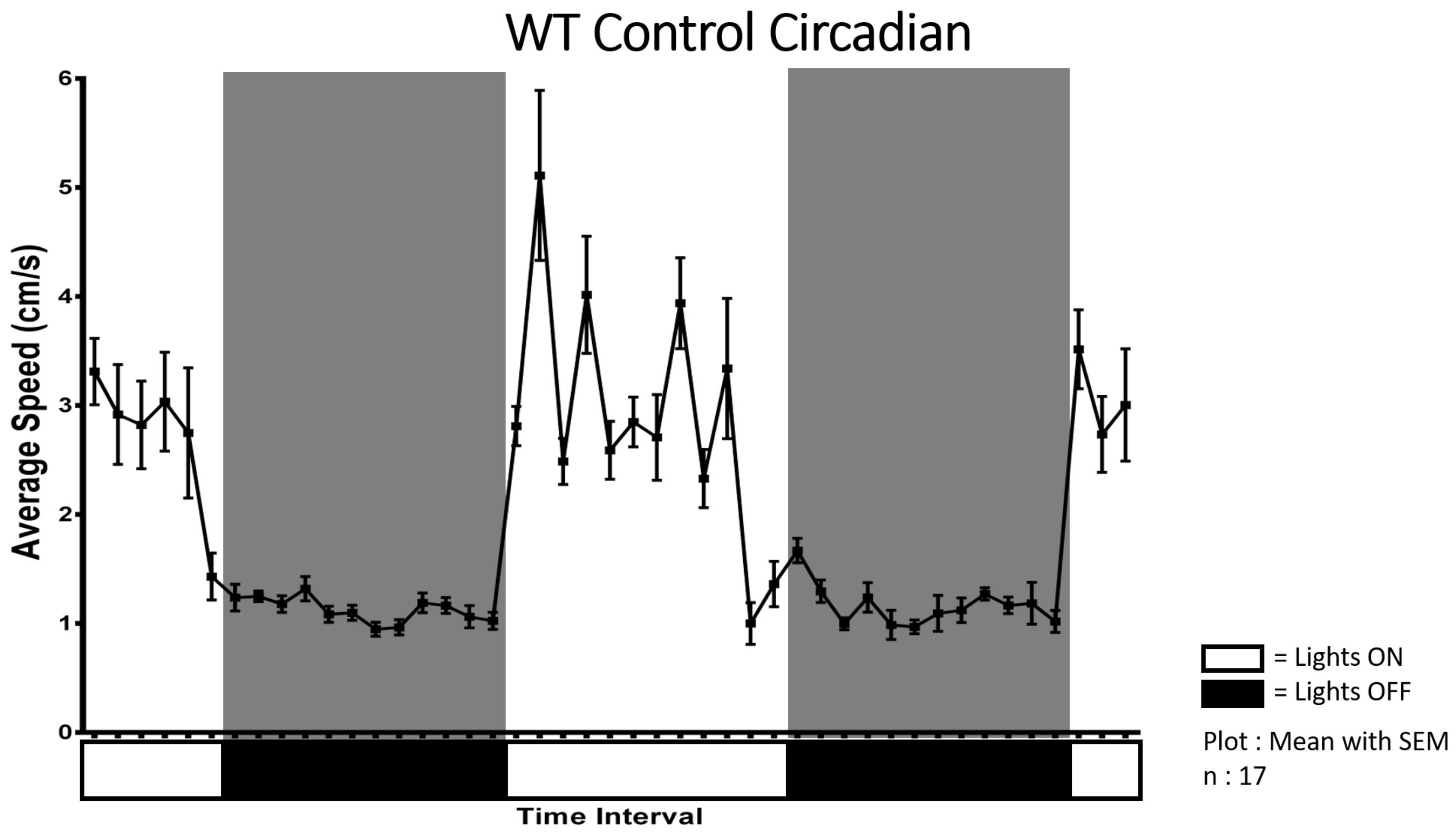

Biology Open • Supplementary information

Method 2 : Establish ImageJ-based method to measure circadian rhythm in fish

Required Programs summarized

Total Recorder

VirtualDub

ImageJ

Microsoft Excel

Biology Open (2019): doi:10.1242/bio.041871: Supplementary information

VirtualDub

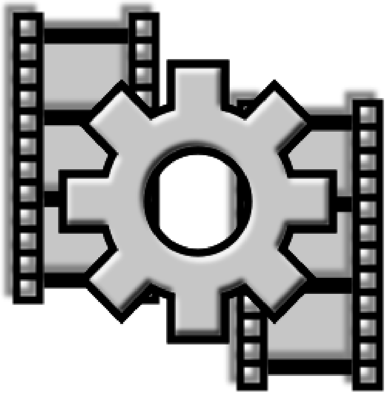

- VritualDub is open source video capture/processing, it is designed to process linear video streams, including filtering and recompression
- The main purpose in this experiment is to convert from video is to recompress the video

Biology Open • Supplementary information

Change Video Output Format in VirtualDub

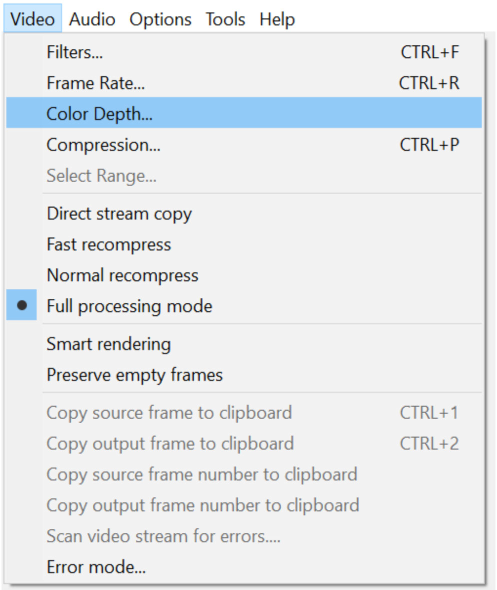

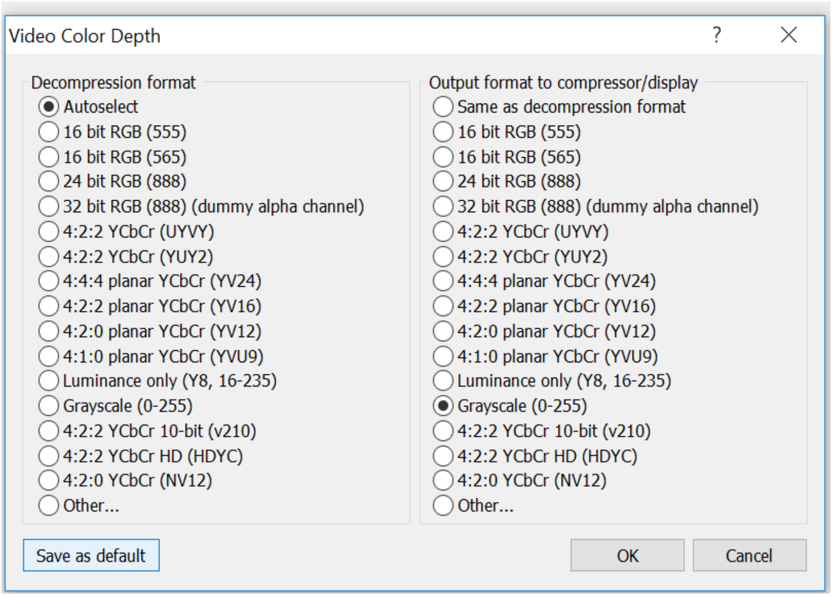

Open the VirtualDub software

Video -> Color Depth

Select output format as 8-bit (Grayscale)

Biology Open • Supplementary information

Open the AVI file in VirtualDub

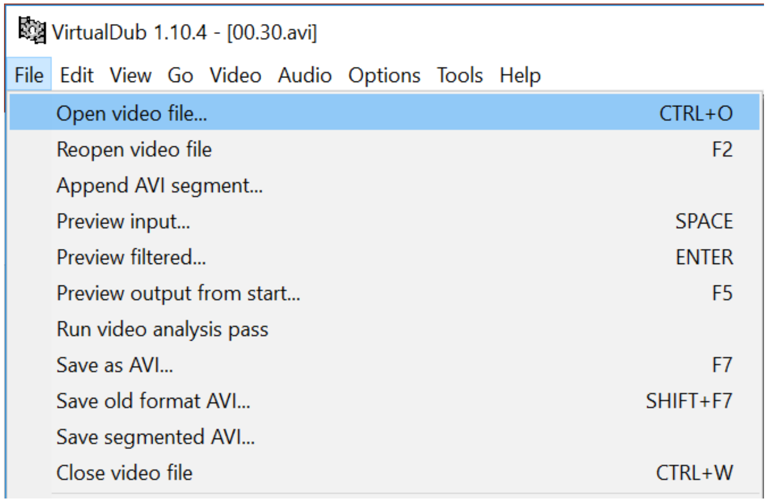

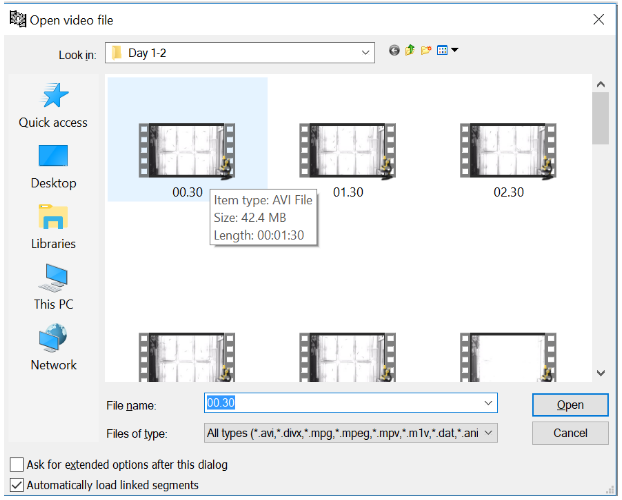

- File -> Open video file (Ctrl+O)
- Select the video file (.avi format)

Biology Open • Supplementary information

Biology Open (2019): doi:10.1242/bio.041871: Supplementary information

Save the Output of VirtualDub

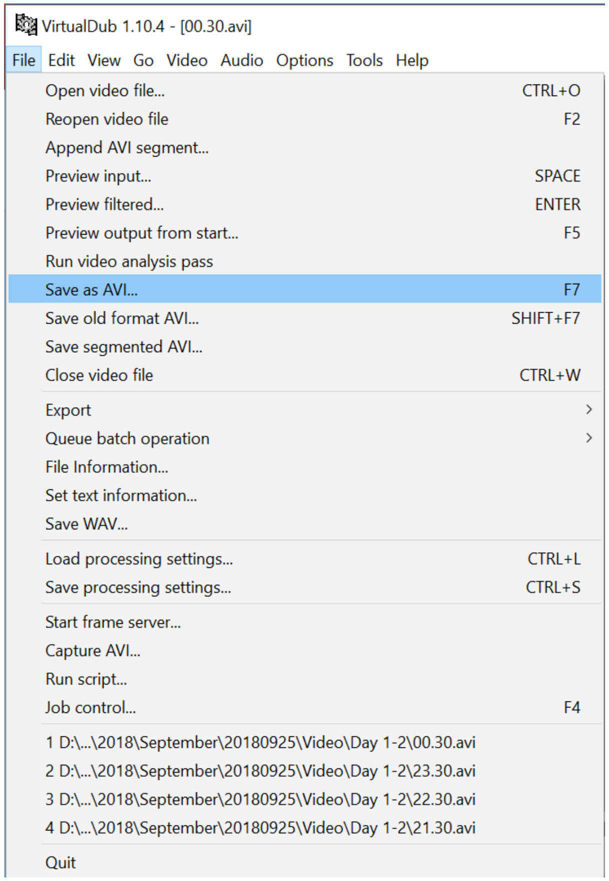

- File -> Save as AVI (F7)

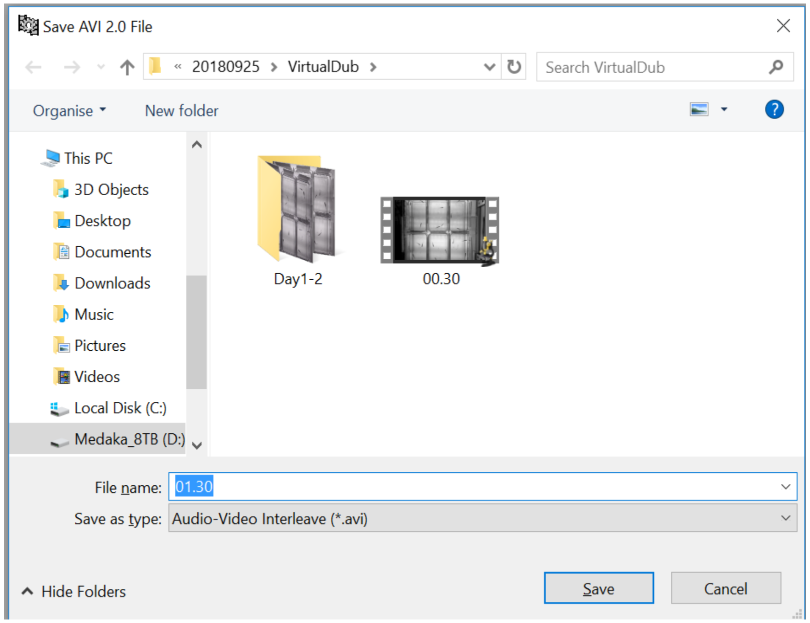

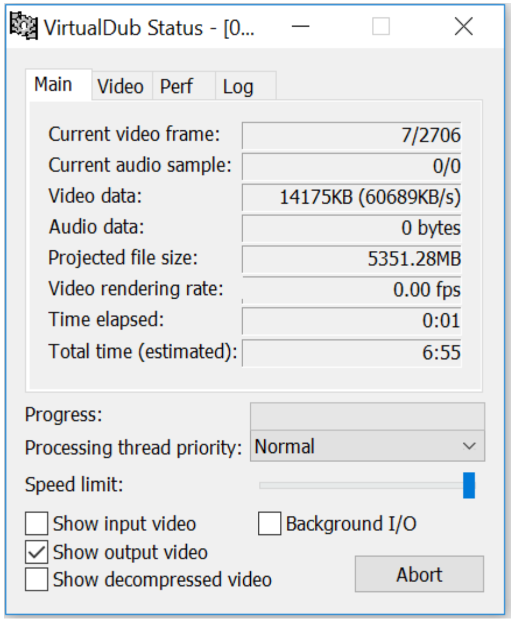

ImageJ

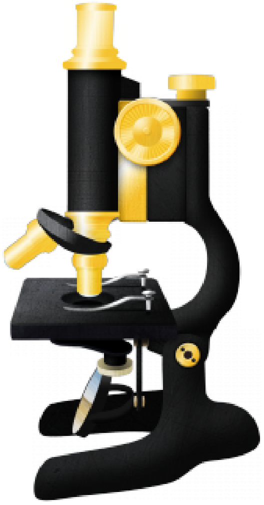

- ImageJ is open source Java-based image processing. It can display, edit, analyse, process, and save images
- The main purpose in this experiment is to analyse the pixel value change in the video
- This platform supported by many plugins that ease the analysis

Open VirtualDub Video in ImageJ

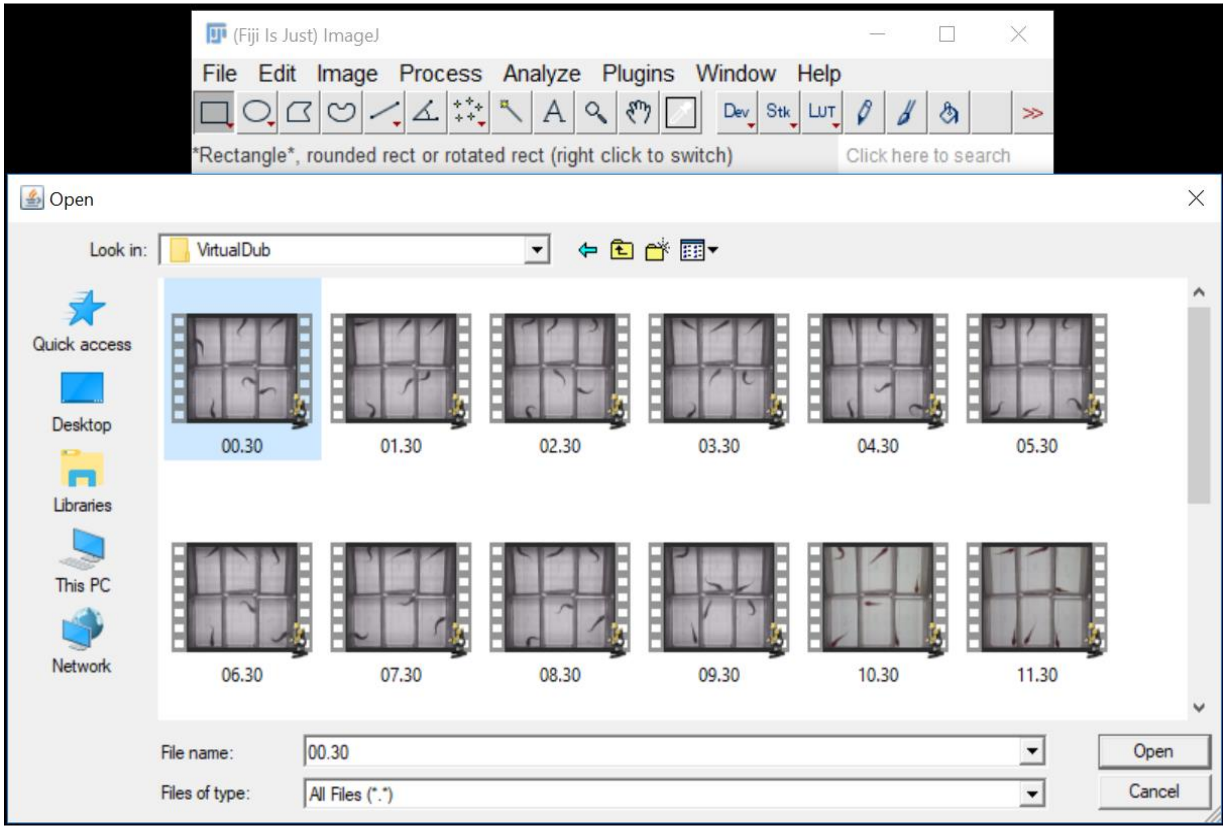

- Open the VirtualDub file

Biology Open • Supplementary information

Convert Video Image Into Greyscale

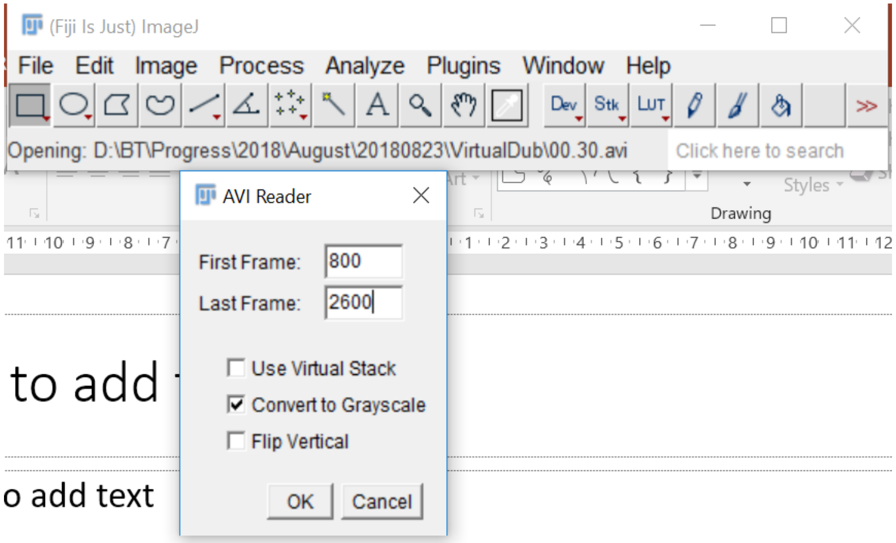

- Select the interested frames
- Convert the VirtualDub into grayscale (8-bit)

Biology Open • Supplementary information

Crop the Interested Area

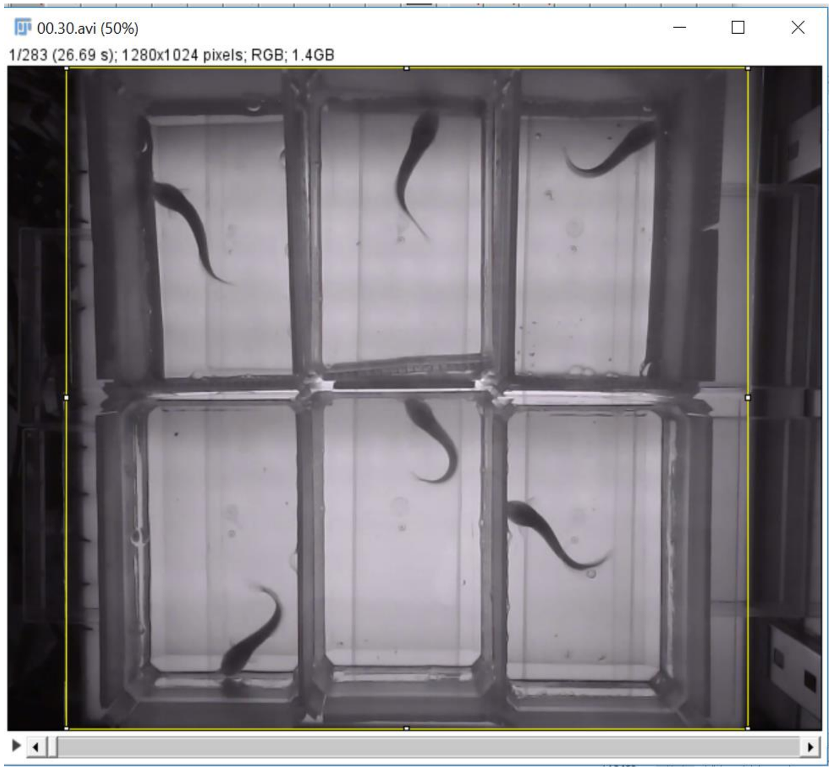

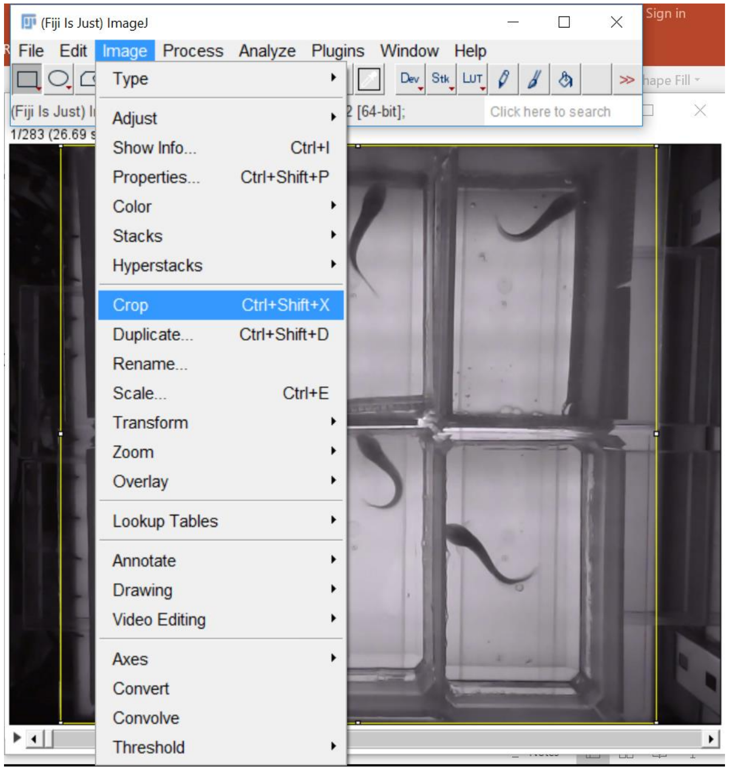

- Select the tanks with rectangle selection
- Crop the area: Image -> Crop (Ctrl+Shift+X)

Duplicate the Video

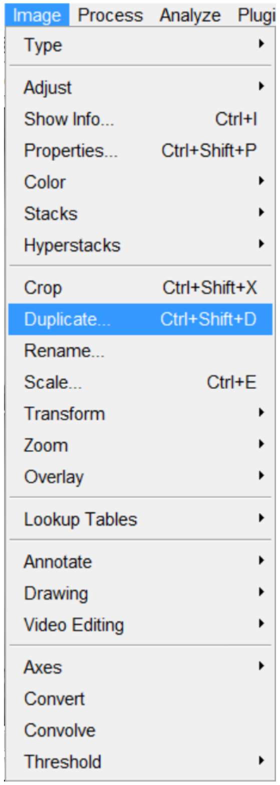

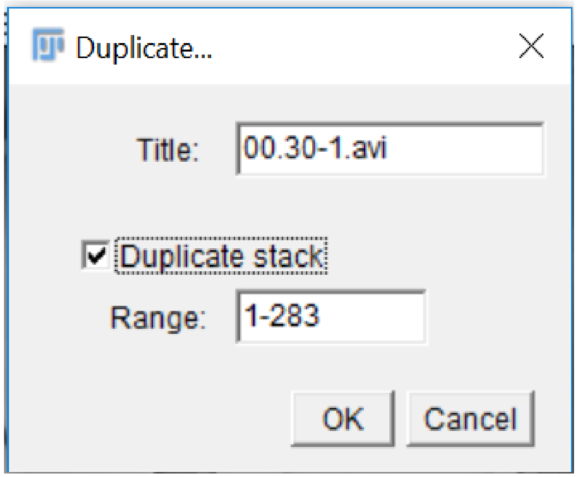

- Duplicate the file to ease the analysis
- Image -> Duplicate (Ctrl+Shift+D) -> Duplicate stack

Biology Open • Supplementary information

Adjust the Threshold

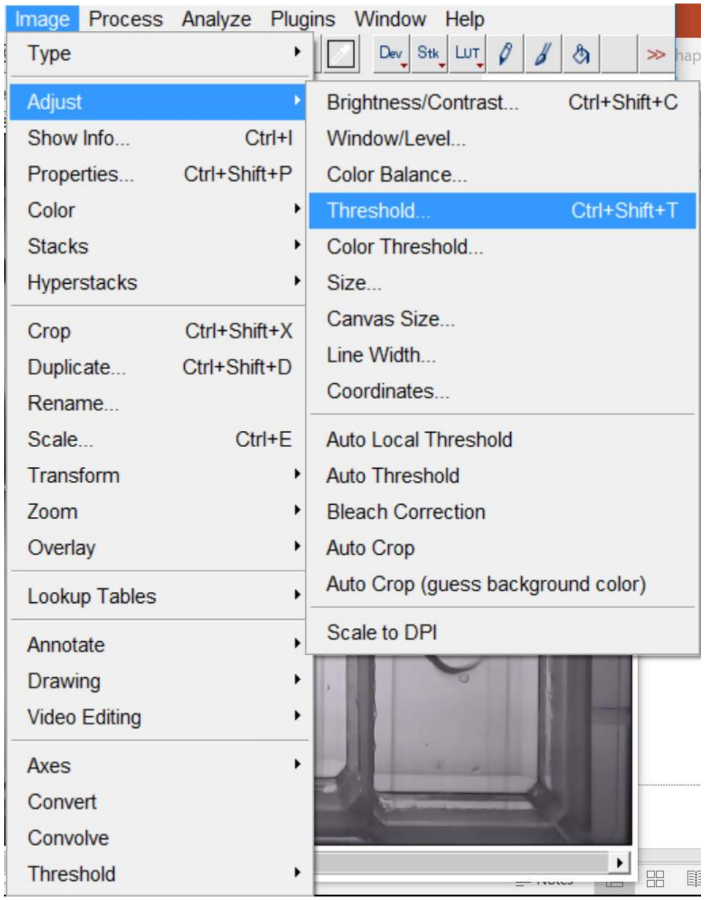

- Adjust the threshold
  Image -> Adjust -> Threshold
  (Ctrl+Shift+T)
- Select the dark background

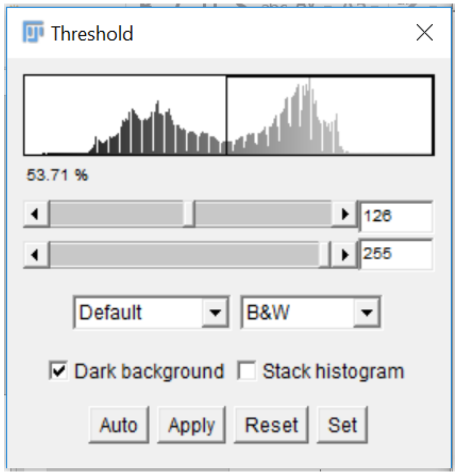

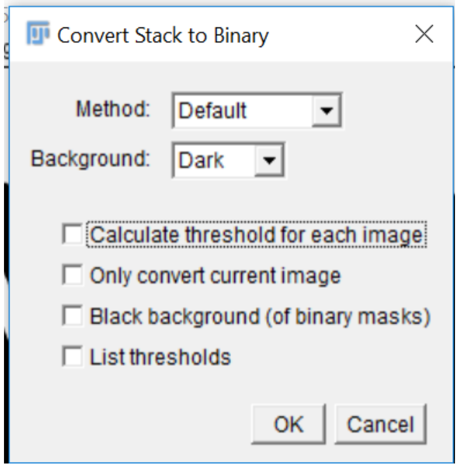

Biology Open • Supplementary information

Use Stack Difference Plugin

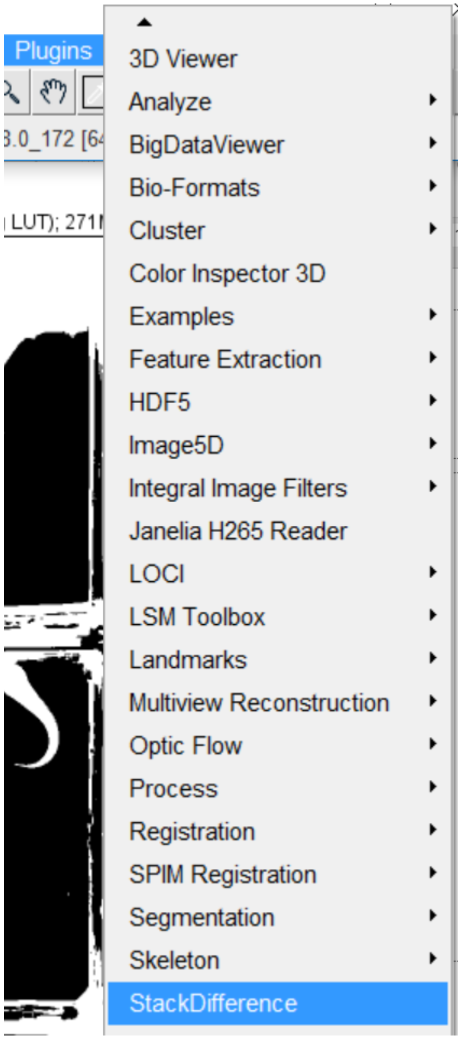

- Stack Difference to see the only object that move (have pixels changed)
- Use stack difference
  Plugins -> StackDifference

Use the Original Video as Template

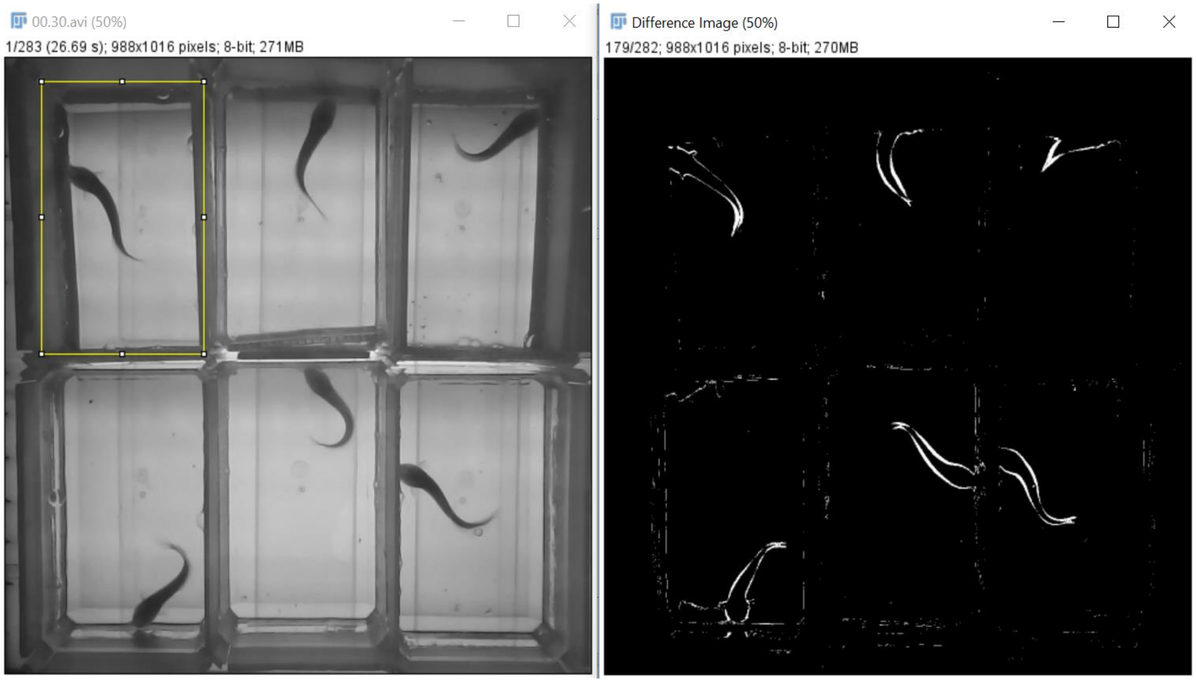

Select the interested area in original VirtualDub file

Restore Selection from the Original Video

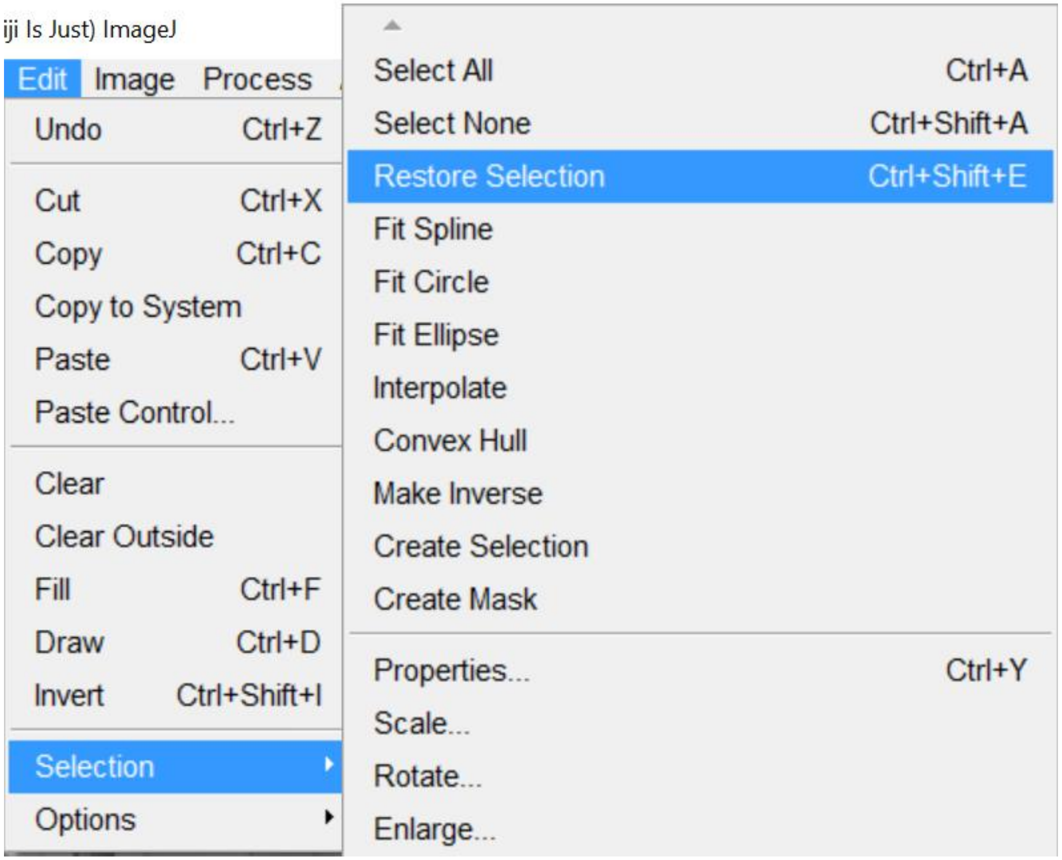

- Click on the duplicated VirtualDub file
- Restore the selection from original file
  Edit -> Selection -> Restore Selection (Ctrl+Shift+E)

Biology Open • Supplementary information

Use Time Series Analyzer Plugin

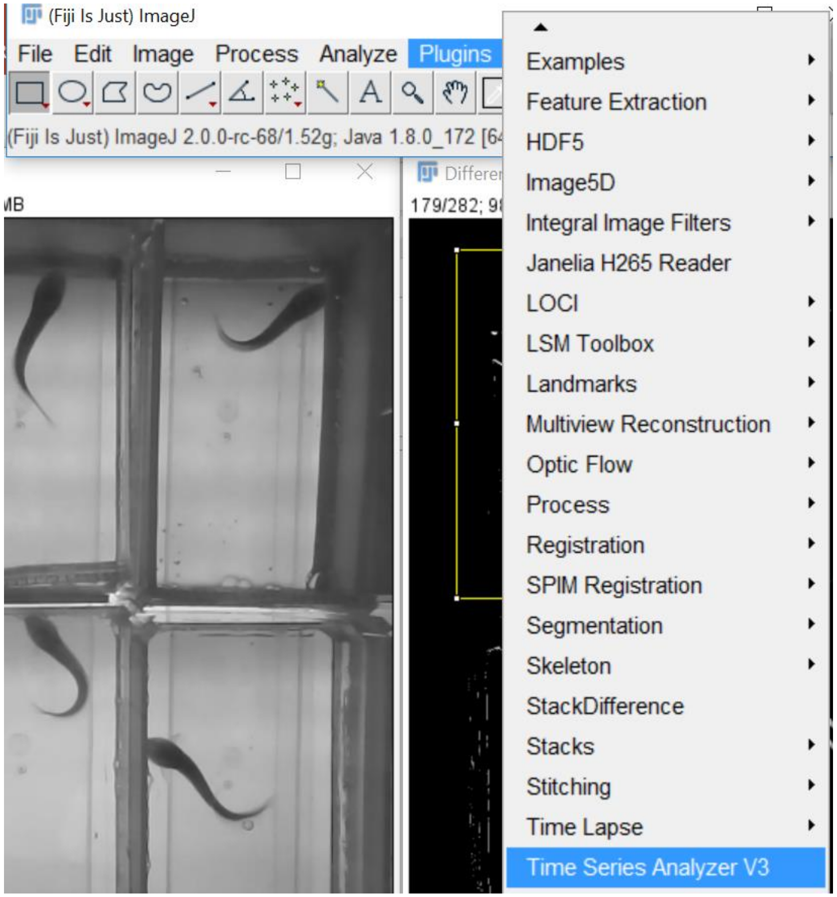

- Use Time Series Analyzer
  Plugins -> Time Series Analyzer V3

Get Average of Pixel Intensity Change

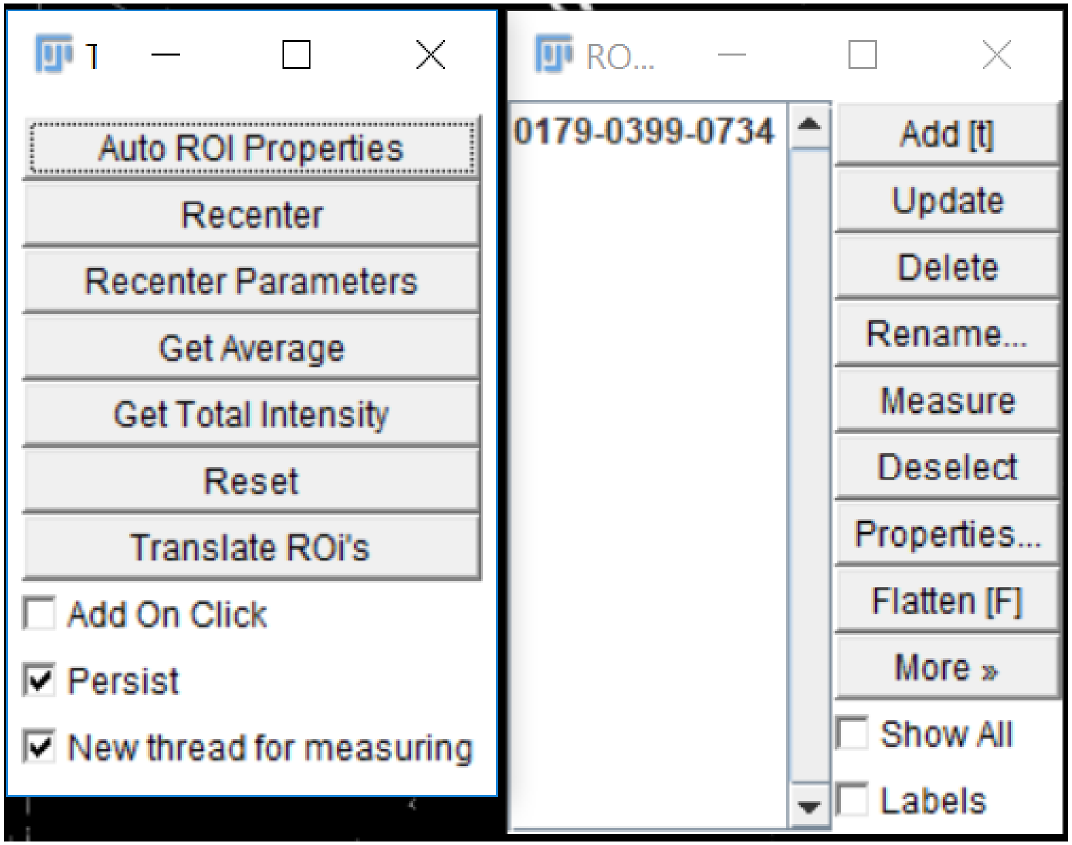

- Use Time Series Analyzer Add -> Get Average

Biology Open • Supplementary information

Get The Dynamic Pixel Change Data Over Time

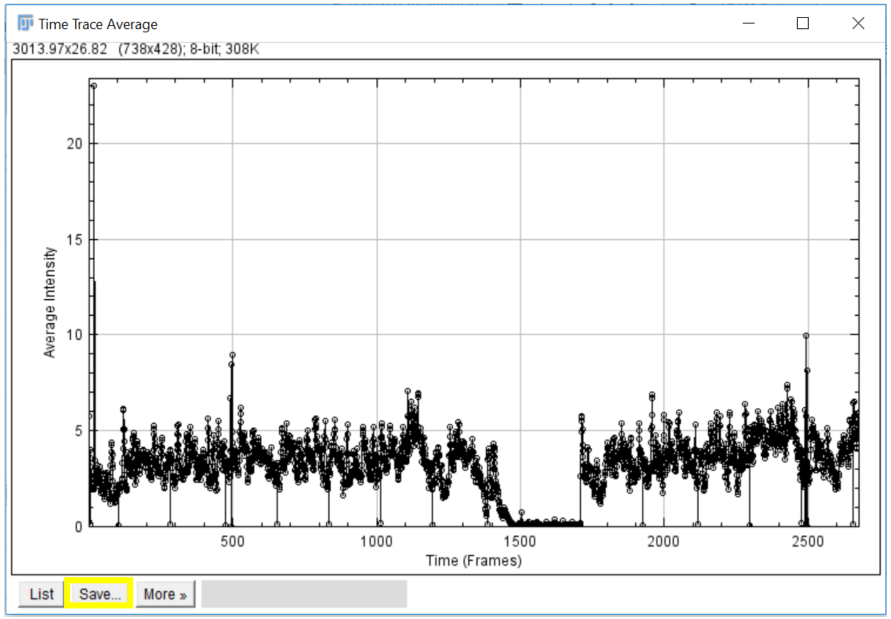

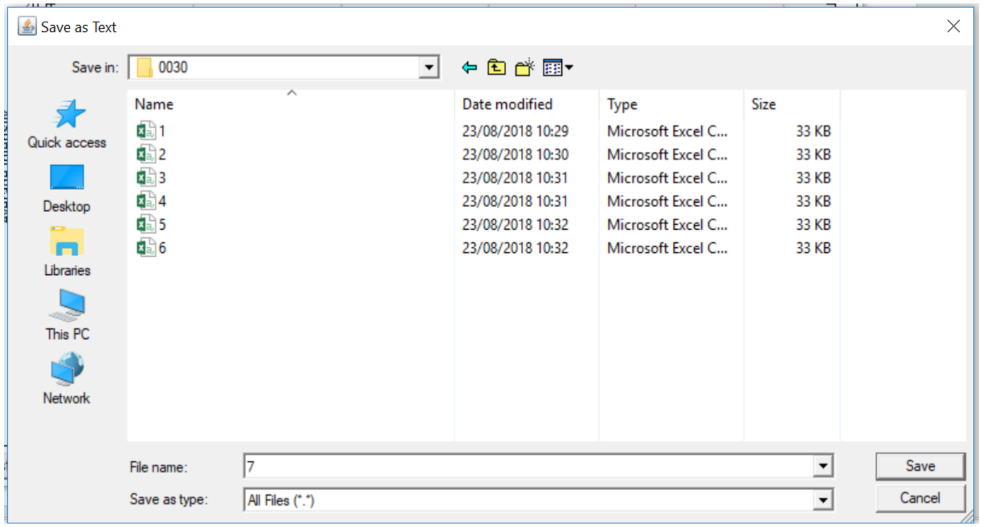

Save the file

Biology Open • Supplementary information

Record Macro can speed up calculation

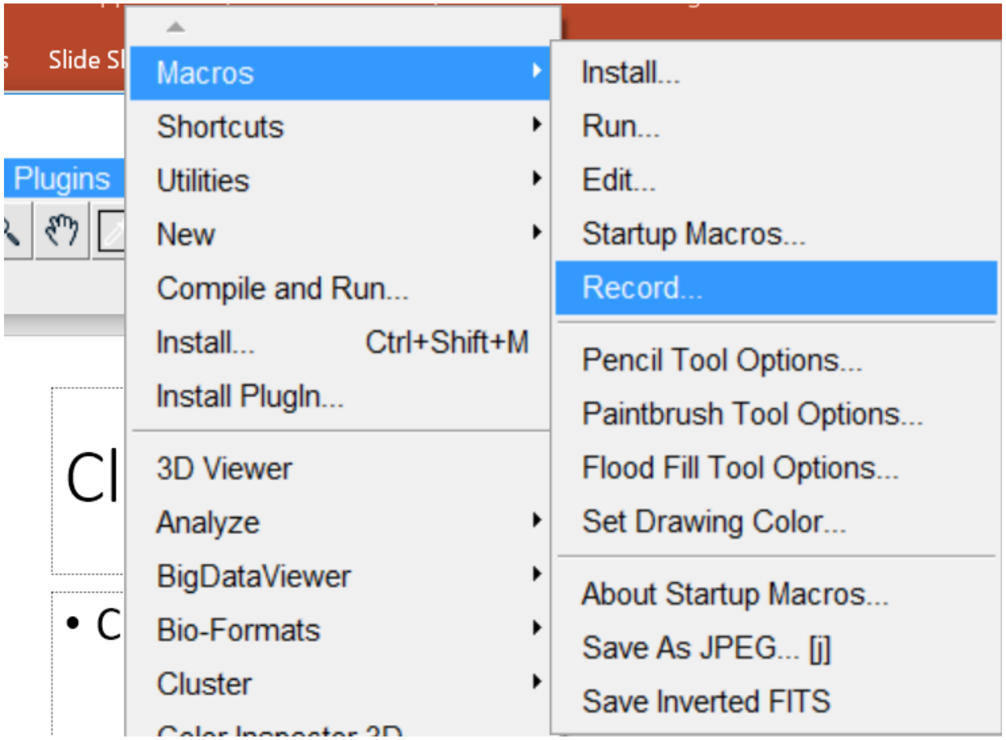

- The macro recorded based on the mouse click
- Plugins -> Macros -> Record

Biology Open • Supplementary information

Record the Features into Macro

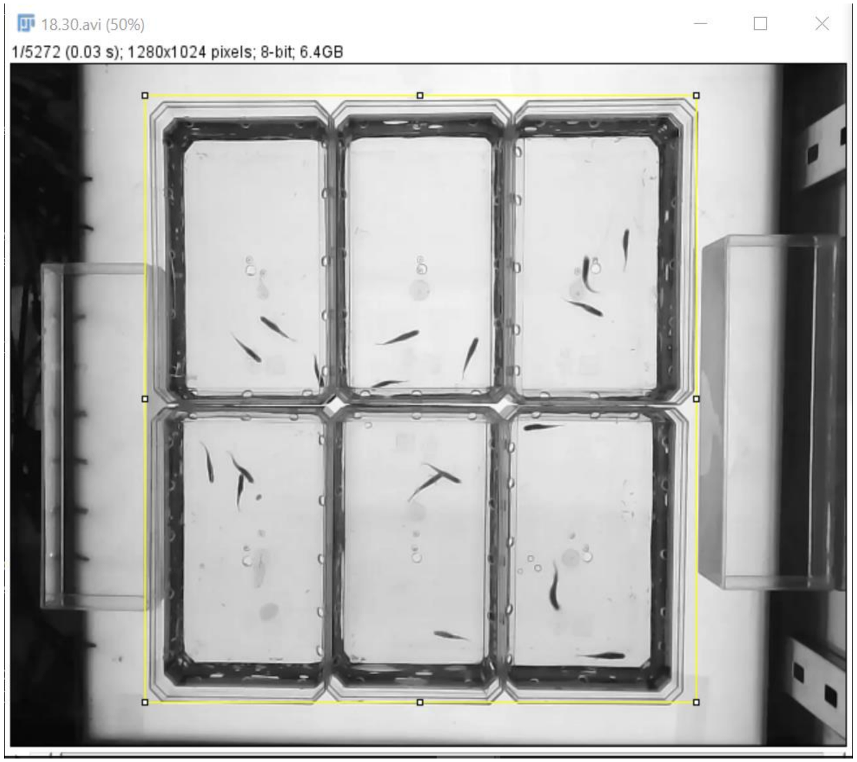

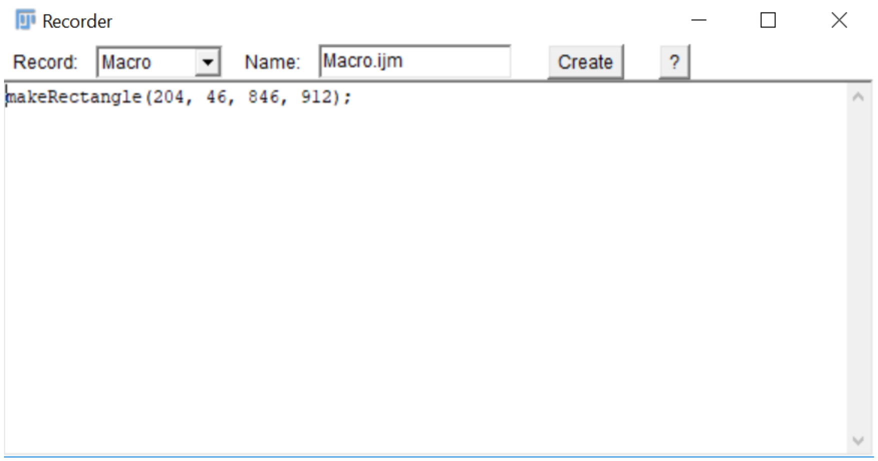

ROI must be adjusted when new experiment conducted with position change

Biology Open • Supplementary information

Create the Macro

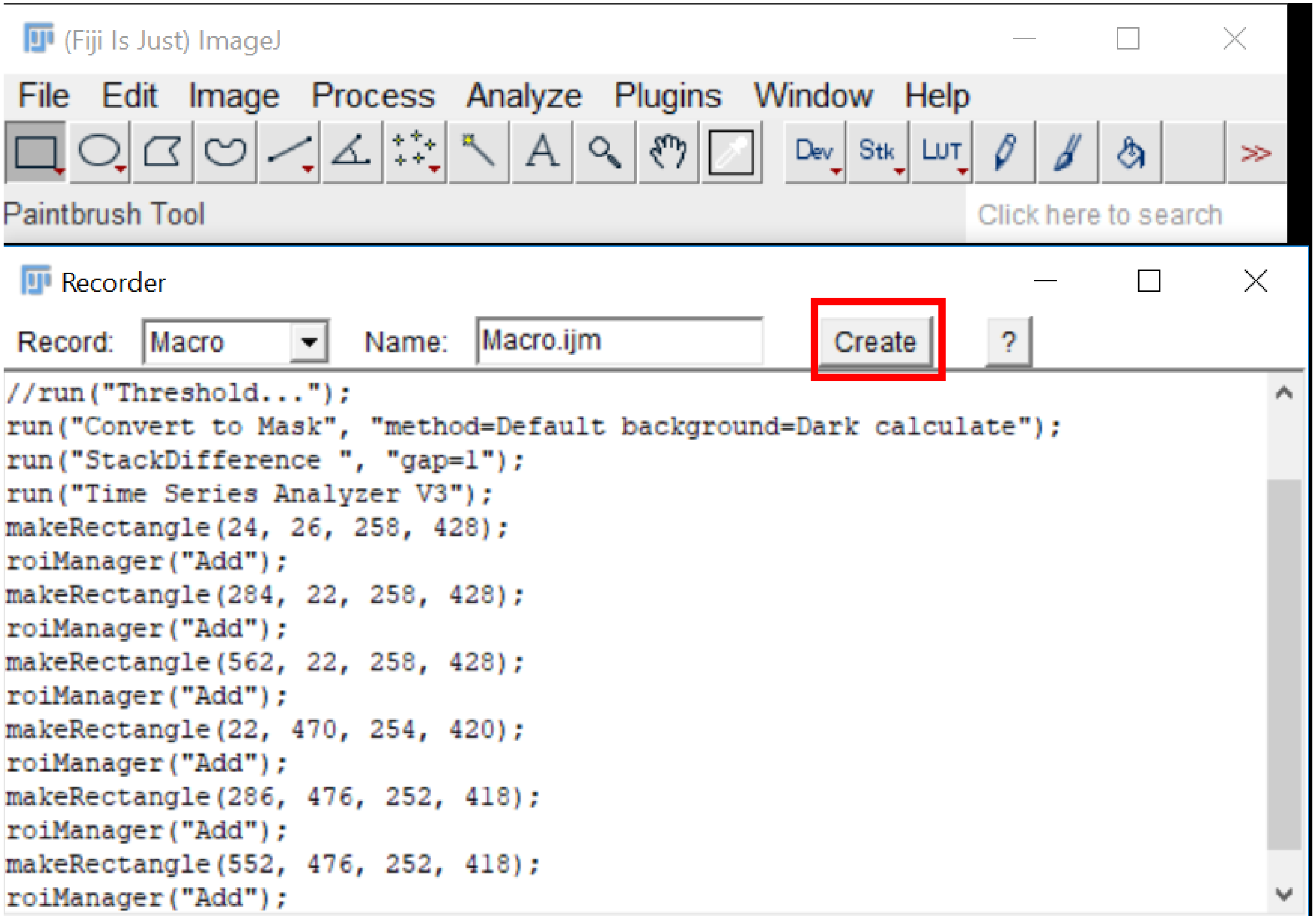

Biology Open • Supplementary information

Save the Macro

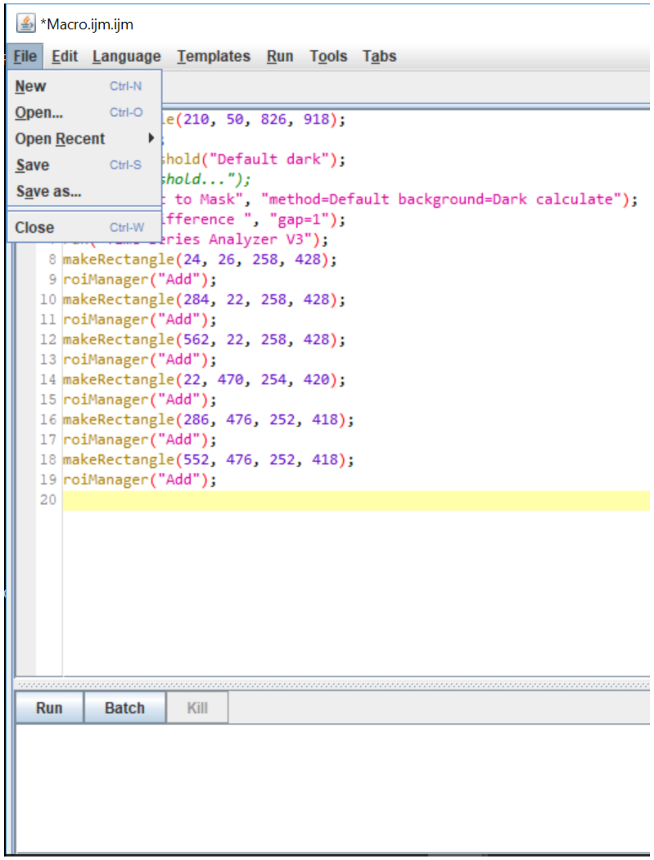

- File -> Save (Ctrl+S)
- Save the macro as .ijm extension

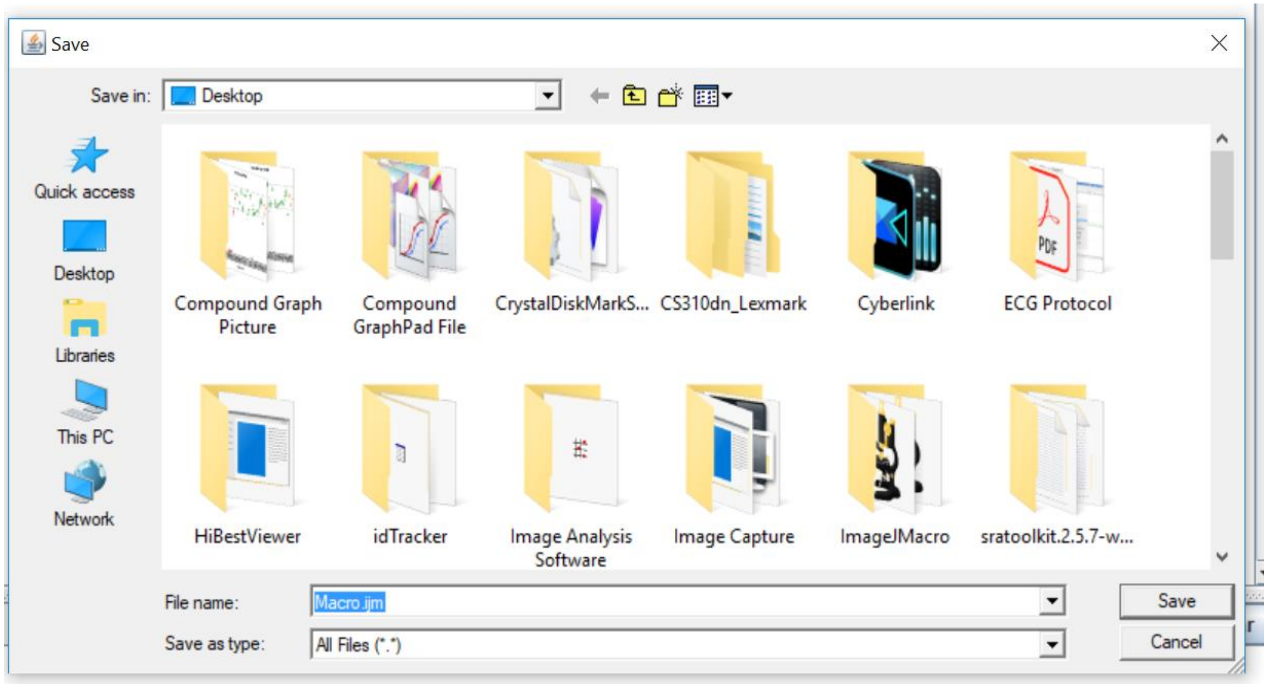

Biology Open • Supplementary information

Biology Open (2019): doi:10.1242/bio.041871: Supplementary information

Run the Macro

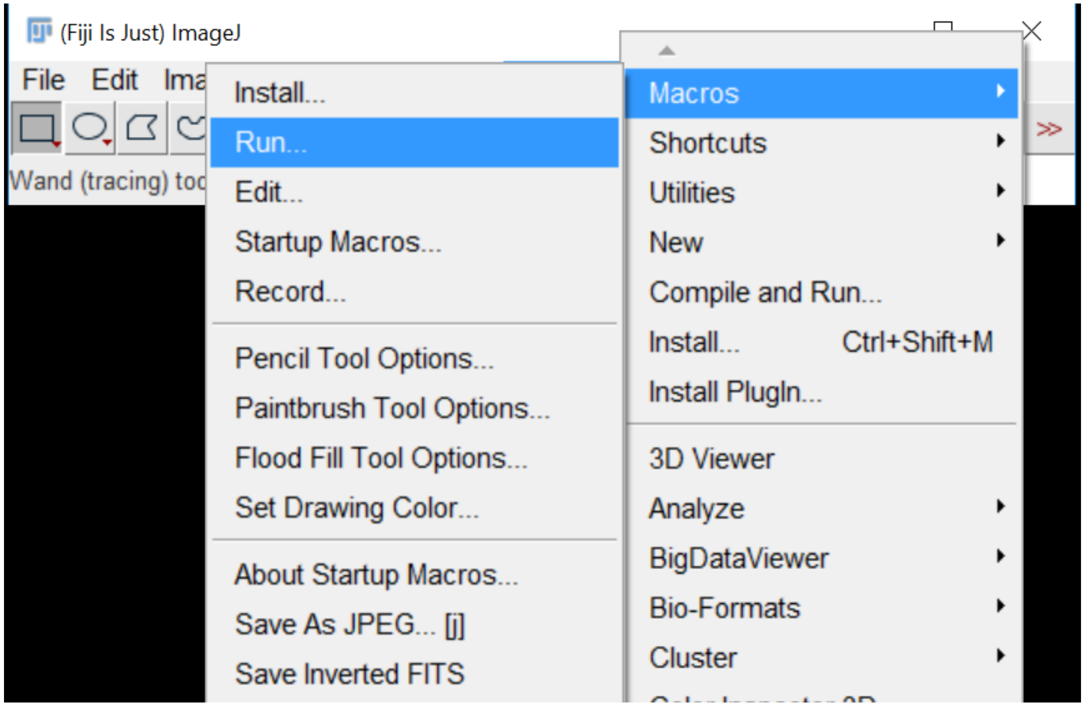

- Plugins -> Macro -> Run

Biology Open • Supplementary information

Biology Open (2019): doi:10.1242/bio.041871: Supplementary information

Select All Regions

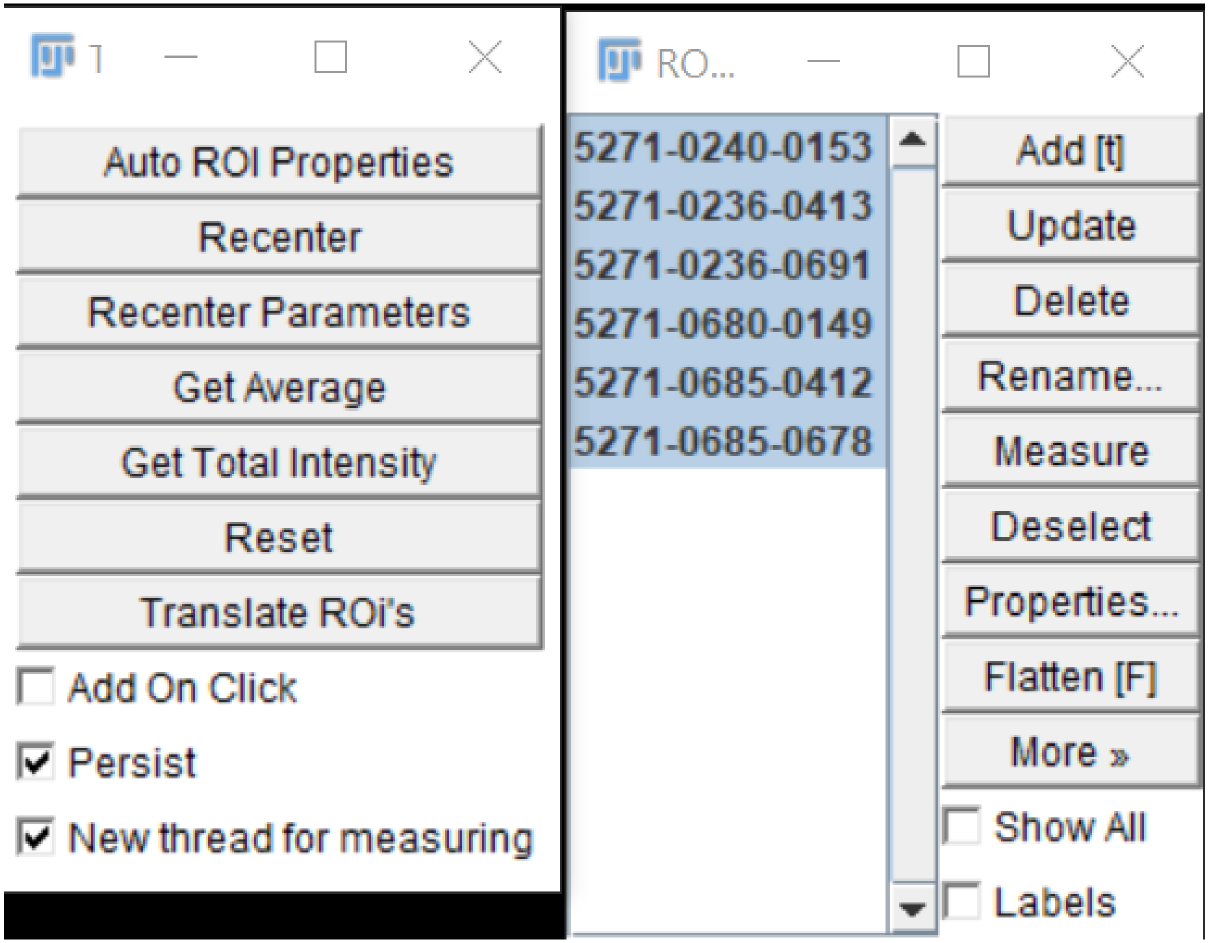

Biology Open • Supplementary information

Copy the Result

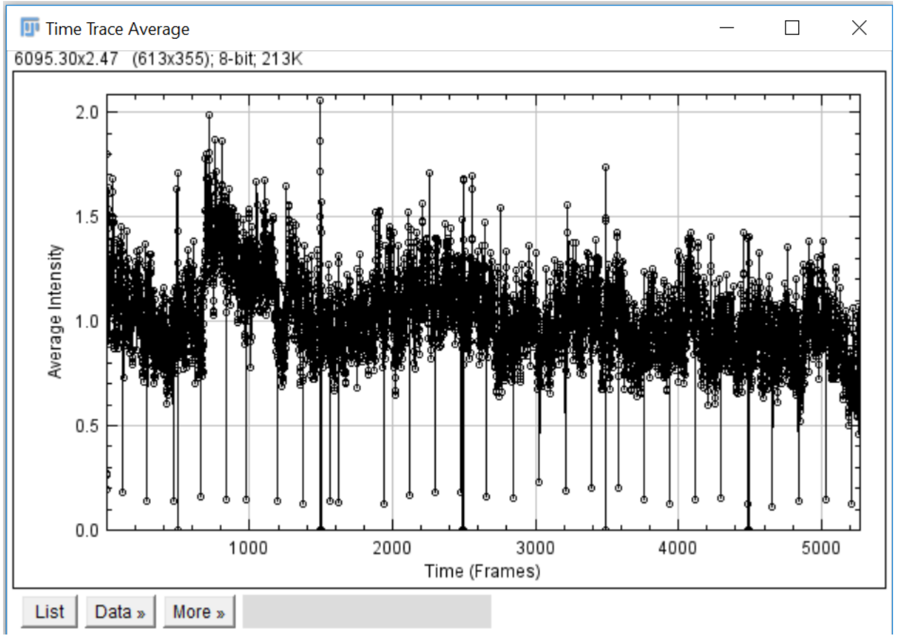

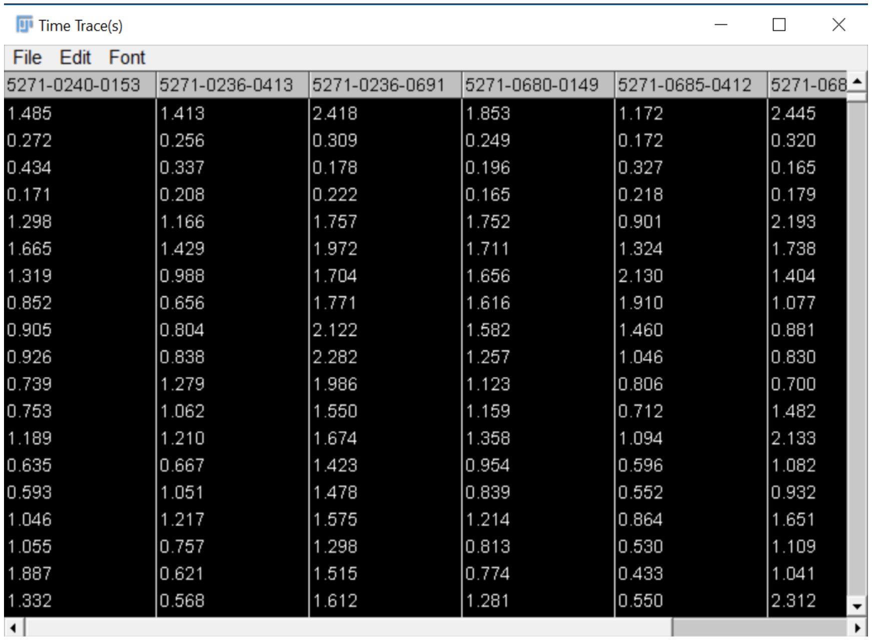

5271-0240-0153	5271-0236-0413	5271-0236-0691	5271-0680-0149	5271-0685-0412	5271-068
1.485	1.413	2.418	1.853	1.172	2.445
0.272	0.256	0.309	0.249	0.172	0.320
0.434	0.337	0.178	0.196	0.327	0.165
0.171	0.208	0.222	0.165	0.218	0.179
1.298	1.166	1.757	1.752	0.901	2.193
1.665	1.429	1.972	1.711	1.324	1.738
1.319	0.988	1.704	1.656	2.130	1.404
0.852	0.656	1.771	1.616	1.910	1.077
0.905	0.804	2.122	1.582	1.460	0.881
0.926	0.838	2.282	1.257	1.046	0.830
0.739	1.279	1.986	1.123	0.806	0.700
0.753	1.062	1.550	1.159	0.712	1.482
1.189	1.210	1.674	1.358	1.094	2.133
0.635	0.667	1.423	0.954	0.596	1.082
0.593	1.051	1.478	0.839	0.552	0.932
1.046	1.217	1.575	1.214	0.864	1.651
1.055	0.757	1.298	0.813	0.530	1.109
1.887	0.621	1.515	0.774	0.433	1.041
1.332	0.568	1.612	1.281	0.550	2.312

- Select all the result
- Copy and paste into the excel file

Biology Open • Supplementary information

Comparison of idTracker- and ImageJ-based method to measure circadian rhythm in fish

	idTracker	ImageJ
Programming language based	Matlab	Java
Endpoint output	More representative	Only in pixel intensity changes
Occluded fish analysis	Hard to analyse	Easy to analyse
Built in Macro for Automatization	Not Available	Available
Analysis time needed (6 tanks, @1 fish)	5'35"	3'(without macro), 2'(with macro)
Analysis time needed (6 tanks, @3 fish)	13"	3'(without macro), 2'(with macro)
Video format supported	Many format	Only AVI
Size of the fish dependency	No	Yes
Individual fish tracking	Able	Not able
Dead fish / noise	Can be excluded	Possible to be counted
Data storage consumption size (24 videos analysis size)	Smaller (~15.5Gb)	Bigger (~349Gb)
Trajectories	Able	Not able
RAM consumption	10 Gb	4 Gb
CPU Usage	75% @3.3GHz	10% @3.3GHz

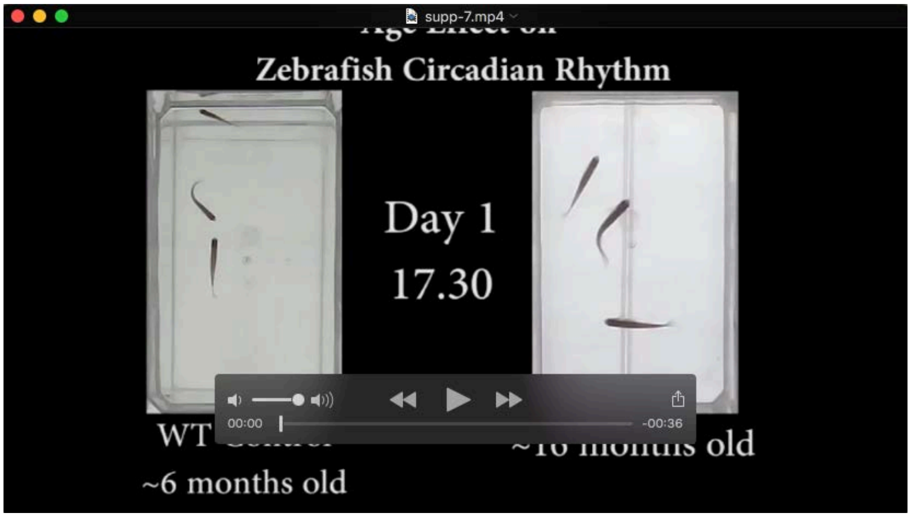

Movie 1. Comparison of circadian locomotion activity between zebrafish and catfish. This video is played at 5x faster speed.

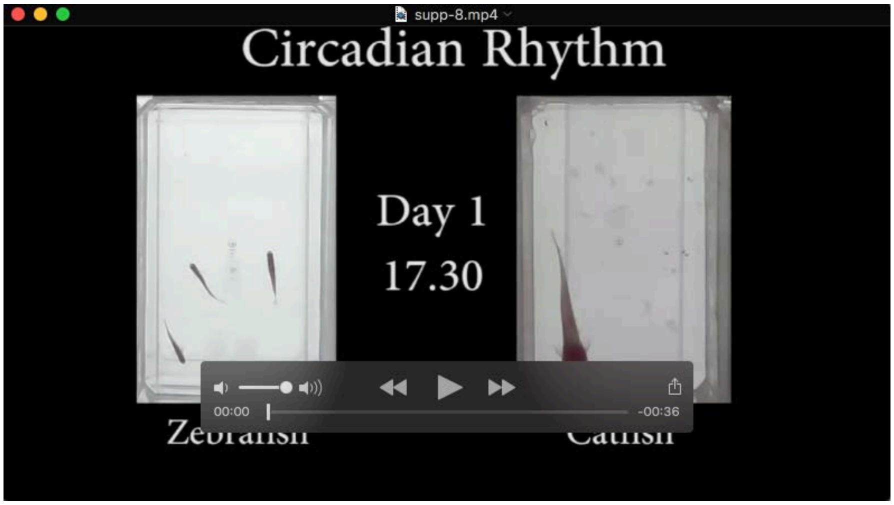

Movie 2. Comparison of circadian locomotion activity between younger (6 month old) and elder (16 month old) zebrafish. This video is played at 5x faster speed.

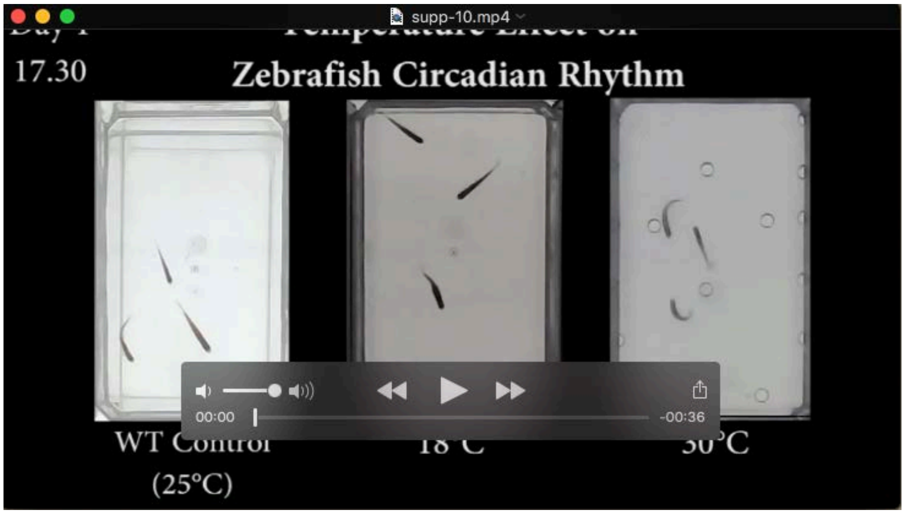

Movie 3. Comparison of circadian locomotion activity for zebrafish acclimated at different ambient temperature of 18, 25 and 30°C. This video is played at 5x faster speed.

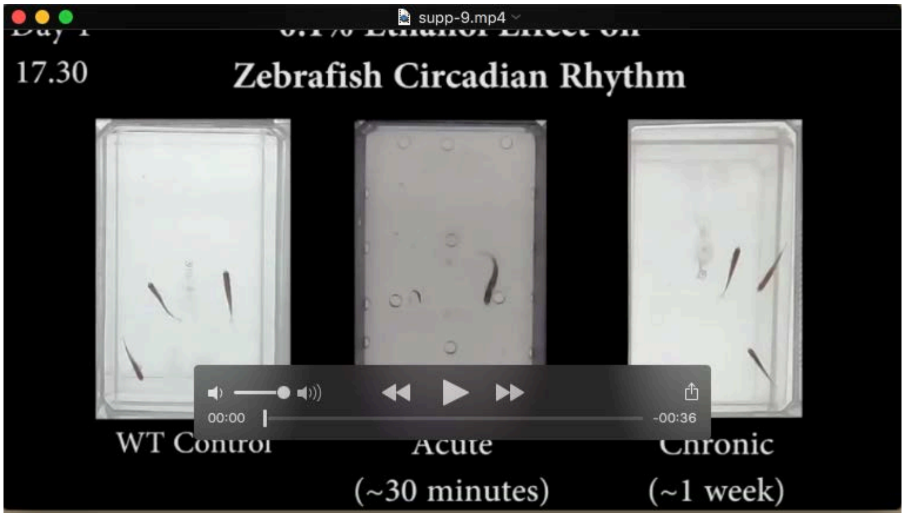

Movie 4. Comparison of circadian locomotion activity for zebrafish exposed to 0.1% ethanol for 30 min (acute) or one week (chronic). This video is played at 5x faster speed.
